# Abiotic Stresses Antagonize the Rice Defence Pathway through the Tyrosine-Dephosphorylation of OsMPK6

**DOI:** 10.1371/journal.ppat.1005231

**Published:** 2015-10-20

**Authors:** Yoshihisa Ueno, Riichiro Yoshida, Mitsuko Kishi-Kaboshi, Akane Matsushita, Chang-Jie Jiang, Shingo Goto, Akira Takahashi, Hirohiko Hirochika, Hiroshi Takatsuji

**Affiliations:** Disease Resistant Crops Research Unit, National Institute of Agrobiological Sciences, Tsukuba, Ibaraki, Japan; Michigan State University, UNITED STATES

## Abstract

Plants, as sessile organisms, survive environmental changes by prioritizing their responses to the most life-threatening stress by allocating limited resources. Previous studies showed that pathogen resistance was suppressed under abiotic stresses. Here, we show the mechanism underlying this phenomenon. Phosphorylation of WRKY45, the central transcription factor in salicylic-acid (SA)-signalling-dependent pathogen defence in rice, via the OsMKK10-2–OsMPK6 cascade, was required to fully activate WRKY45. The activation of WRKY45 by benzothiadiazole (BTH) was reduced under low temperature and high salinity, probably through abscisic acid (ABA) signalling. An ABA treatment dephosphorylated/inactivated OsMPK6 via protein tyrosine phosphatases, OsPTP1/2, leading to the impaired activation of WRKY45 and a reduction in *Magnaporthe oryzae* resistance, even after BTH treatment. BTH induced a strong *M*. *oryzae* resistance in *OsPTP1/2* knockdown rice, even under cold and high salinity, indicating that OsPTP1/2 is the node of SA-ABA signalling crosstalk and its down-regulation makes rice disease resistant, even under abiotic stresses. These results points to one of the directions to further improve crops by managing the tradeoffs between different stress responses of plants.

## Author Summary

Chemical defence inducers make plants resistant to diseases such as rice blast. However, plants sometimes become more pathogen susceptible under abiotic stresses even in their presence. Because such regulation prioritizes the responses to the most life-threatening stress, it could be necessary for plants to survive in nature. However, it seems dispensable or even disadvantageous for crops cultivated under fertile conditions. Here, we show the molecular mechanism underlying one of such phenomena in rice. WRKY45 is a central transcription factor that regulates strong defence signalling mediated by salicylic acid. We found that WRKY45 is activated through phosphorylation by a protein kinase, OsMPK6, which is activated by dual phosphorylation in response to the defence signalling. We also found that OsMPK6 can be inactivated by tyrosine dephosphorylation in response to abiotic stresses such as low temperature and high salinity probably mediated by abscisic acid, leading to reduction of WRKY45-dependent disease resistance. Moreover, we found that specific tyrosine protein phosphatases dephosphorylate/inactivate OsMPK6 in response to abiotic stresses. Knockdown of their genes rendered rice plants resistant against blast disease even under the abiotic stresses, pointing to the way to further improve rice.

## Introduction

Plants, as sessile organisms, are continuously exposed to various environmental stresses in nature. To cope with such conditions using limited resources, plants have evolved various mechanisms that enable resource allocation to the most life-threatening stress [1] [2]. Such tradeoffs between the responses to different stresses are often regulated by crosstalk between signalling pathways [3] [4] [5]. A number of studies have reported various signalling components that appear to influence signalling crosstalk. However, the precise molecular mechanisms that regulate the crosstalk remain poorly understood in most cases [6] [4] [7].

The salicylic acid (SA) signalling pathway plays a crucial role in pathogen defence. In Arabidopsis, NPR1, the transcriptional cofactor, plays a major role in the SA defence signalling pathway [8]. In rice (*Oryzae sativa*), in addition to OsNPR1/NH1, the rice ortholog of NPR1, the transcription factor (TF) WRKY45 plays a crucial role in the branched SA pathway [9] [10] [11] [12]. Up-regulation of WRKY45 by chemical defence inducers, such as benzothiadiazole (BTH), or its overexpression, renders rice plants resistant against several pathogens, including fungus, such as *Magnaporthe oryzae* causing blast disease, and bacterium, such as *Xanthomonas oryzae* pv. *oryzae* causing leaf blight disease [9] [13] [14], without major negative effects on plant growth. WRKY45 auto-regulates the transcription of its own gene [12] and is regulated by the ubiquitin-proteasome system [15].

Abscisic acid (ABA) signalling is mainly involved in plant responses to abiotic stresses, such as the cold, drought, and high salinity [16] [17]. However, ABA also acts as a modulator of defence responses against pathogens, both positively and negatively, with its negative role being more prevalent [18] [3] [19] [20] [4] [21] [5] [22]. Recent studies have shown that ABA antagonizes SA-signalling, thereby interfering with defence responses in tomato, Arabidopsis, and rice [23] [24] [25].

The WRKY TFs can be phosphorylated and activated by MAP kinases, as is the case with Arabidopsis WRKY33 [26] and *Nicotiana benthamiana* WRKY8 [27]. The negative regulation of MAP kinases through dephosphorylation by protein phosphatases, including Ser/Thr-specific phosphatases, dual-specificity phosphatases, and Tyr-specific phosphatases (PTPases), has been reported [28] [29]. We have previously reported that activated MAP kinases can phosphorylate WRKY45 *in vitro*, and MAP kinases can be activated in response to SA [30]. However, details of WRKY45 phosphorylation, and the biological significance of the phosphorylation have remained unknown.

Rice blast is one of the most serious crop diseases in the world. It has been reported that rice plants are more blast susceptible under abiotic stresses, such as low temperature and drought [31] [32] [33]. In rice, ABA treatment severely compromised *M*. *oryzae* resistance [34], which is mediated by suppression of *WRKY45* and *OsNPR1/NH1* genes via ABA signalling [25] [35]. These authors suggest that ABA signalling plays a role in increased blast susceptibility under low temperature.

In this report, we show the mechanism underlying blast resistance through the activation of WRKY45 by MAP kinase (MAPK)-dependent phosphorylation in the SA pathway. Moreover, we showed that the tyrosine dephosphorylation of the MAPK by PTPases, OsPTP1/2, is responsible for the MAPK inactivation under abiotic stresses or in the presence of exogenous ABA. Additionally, the knockdown of *OsPTP1/2* uncoupled the induced blast resistance from the abiotic stresses. These findings should enable the development of technologies to protect rice from diseases even under the influence of environmental factors.

## Results

### Phosphorylation is required for full activation of WRKY45

WRKY45 was phosphorylated by OsMPK6 *in vitro* in the presence of a constitutively active form of OsMKK10-2 (MKK10-2D, Os03g0225100, LOC_Os03g12390.1), which is a rice MAPK kinase that phosphorylates and activates OsMPK6 *in vitro* [30]; however, its biological significance was unknown. To further analyse the WRKY45 phosphorylation, we therefore determined the sites in WRKY45 that are phosphorylated by OsMPK6. Incubation *in vitro* of fragmented WRKY45 polypeptides fused to a maltose binding protein (MBP) with OsMPK6 revealed that only two regions (amino acids 239–292 and 281–326) of WRKY45 were phosphorylated (S1 Fig). By a consequent substitution study on candidate phosphorylation sites (Ser or Thr), we found that Thr^266^, Ser^294^, and Ser^299^ were required for phosphorylation of WRKY45 by OsMPK6 *in vitro* (S2 Fig).

**Fig 1:**
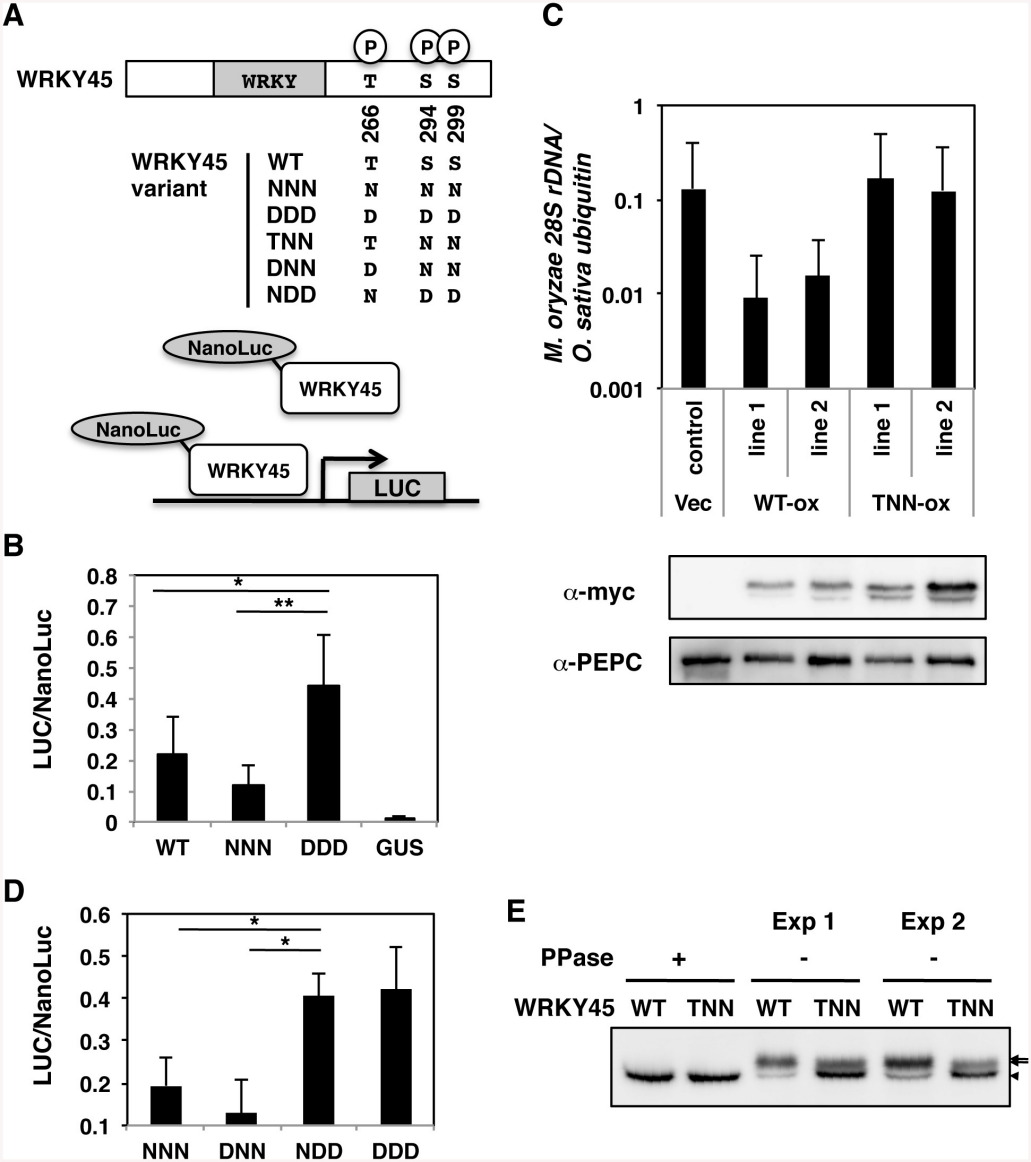
Phosphorylation at carboxyl-terminal serines activates WRKY45 in rice. (A) Schematic representation of WRKY45 variants and the reporter assay system. Luciferase activities, based on the transactivation by NanoLuc-WRKY45 fusion proteins per total NanoLuc activity, were determined as specific transactivation activities. N and D represent dephosphomimetic (Asn) and phosphomimetic (Asp) substitutions at the Thr^266^, Ser^294^, and Ser^299^of the WRKY45 proteins. (B) Specific transactivation activities of wild-type (WT) and mutant WRKY45. The mutants in which the three amino acids were changed to N (NNN) or D (DDD) simultaneously were assayed as described in the Methods. Means of three or more biological replicates are shown with SD. *, *P* < 0.02; **, *P*<0.002 (Student’s *t*-test). (C) Blast resistance assay of myc-tagged WT and mutant *WRKY45*-overexpressing (ox) rice plants. Blast resistance was evaluated by quantifying *M*. *oryzae 28S rDNA* using genomic qPCR. Means of three or more biological replicates are shown with SD. Accumulation of the WT and mutant WRKY45 proteins, in which the two serines were substituted with Asn (TNN), were detected by anti-myc antibody. Phosphoenolpyruvate carboxylase (PEPC) was immunodetected as the loading control. (D) Specific transactivation activities of DNN and NDD mutants of WRKY45, in which the three amino acids were independently changed to N or D. The assays were performed similarly to those in (B). (E) Immunodetection of the WT and TNN mutant of WRKY45 proteins in plants was performed after treatment with (+) or without (-) lambda protein phosphatase (PPase) *in vitro*. Two independent sets of experiments were done (Exp 1 and -2). Arrows and an arrowhead indicate phosphorylated and dephosphorylated proteins, respectively. Numbers of bands appear to be different from those in 2C presumably because of different resolution of the gels.

To assess the effects of the phosphorylation on transcriptional activity, we generated mutant forms of WRKY45 in which all the three amino acids were replaced by Asn (NNN) or Asp (DDD) to mimic dephosphorylation and phosphorylation of all three sites, respectively, and tested them by transient reporter assays (Fig 1A). The transactivation activity of DDD was 2–4-fold higher than that of NNN (Fig 1B), indicating that the phosphomimetic mutation elevated the transcriptional activity of WRKY45. These results suggest that WRKY45 phosphorylation results in its activation *in vivo*. The level of wild-type (WT) WRKY45 activity was between those of NNN and DDD (Fig 1B), consistent with its partial phosphorylation state. We attempted to generate transgenic plants overexpressing NNN and DDD mutants tagged with the myc sequence at their N-termini, but we obtained neither of them. Then, we focused on the carboxyl-terminal region that contained two of the three phosphorylation sites, the closely located Ser residues, Ser^294^ and Ser^299^, because the carboxyl-terminal region of WRKY45 is critical for transcriptional activity [15]. We generated mutants in which the two serines were substituted with Asp (TDD) or Asn (TNN), and attempted to overexpress the mutant cDNAs in rice transformants (Fig 1A). While we were unable to obtain the transformants for TDD, we obtained several for TNN. Whereas the TNN transformants accumulated the transgene-derived proteins to levels comparable, or even slightly higher, than those in WT *WRKY45*-overexpressing (ox) transformants, they showed no significant enhancement of blast resistance (Fig 1C), indicating that the substitution of these two serines compromised the function of WRKY45. To examine the contribution of the phosphorylation at these two Ser to transcriptional activity, we generated new mutants, DNN and NDD, and analysed them in the transient system (Fig 1A). The relative activity of NDD was as high as that of DDD, while that of DNN was as low as that of the NNN mutant (Fig 1D). These results suggest that the phosphorylation of Ser^294^ and/or Ser^299^, but not that of Thr^266^, is important for the transcriptional activity of WRKY45, consistent with the results of blast resistance test (Fig 1C).

To examine whether these two serines in WRKY45 are phosphorylated *in vivo*, we treated the extracts from WT and TNN *WRKY45*-ox plants with lambda protein phosphatase (PPase). The electrophoretic mobility of the TNN form treated with the PPase was indistinguishable from that of WT WRKY45 (Fig 1E), indicating that the effect of Ser-to-Asn substitutions at the two sites on the electrophoretic mobility is negligible. Without the PPase treatment, two bands were seen in both WT and TNN extracts (Fig 1E), suggesting that both WT and the mutant WRKY45 proteins were phosphorylated *in vivo*. The slower mobility (upper) bands in WT extracts were broad and much more intense than the faster mobility (lower) band for the unphosphorylated WRKY45 protein. By contrast, the upper band in the TNN extract was less intense than the lower band and thinner. These reproducible results suggest that Ser^294^ and/or Ser^299^ are/is actually phosphorylated in plant cells. This idea was also supported by the results of Phos-Tag polyacrylamide gel electrophoresis [36] (S3 Fig). Taken together, the phosphorylation of Ser^294^ and/or Ser^299^ of WRKY45 and consequent activation of its transcriptional activity are required for the full functioning of WRKY45-dependent blast resistance.

### OsMPK6 was dually phosphorylated in response to SA in rice plants

**Fig 2:**
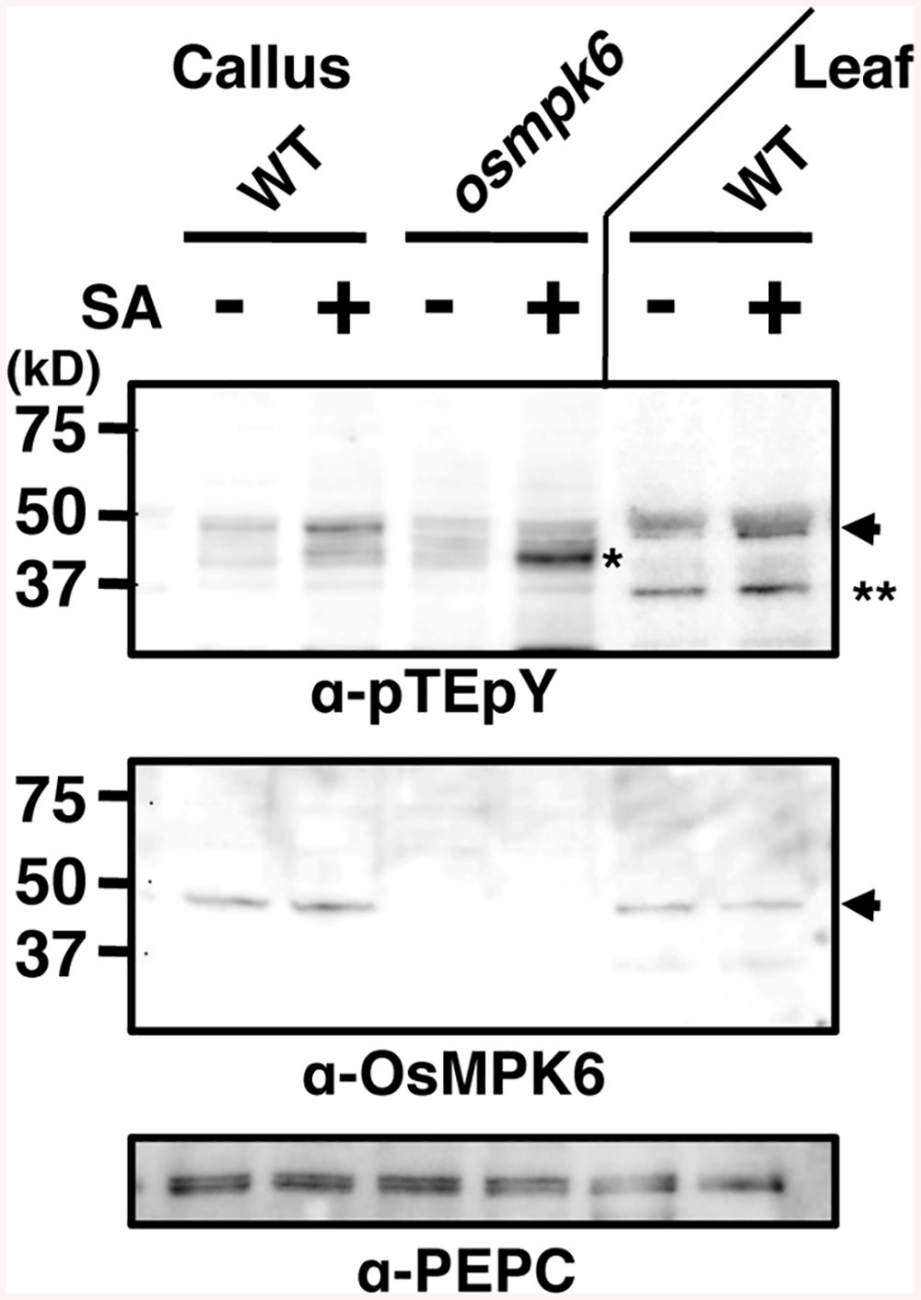
Salicylic acid (SA) induces OsMPK6 phosphorylation. Rice calli or leaves were treated with 1 mM SA, and proteins with a doubly phosphorylated TEY motif were immunodetected with an anti-pTEpY antibody. NB was used as WT leaf source. OsMPK6 and PEPC were detected by specific antibodies. Arrows, OsMPK6; * and **, other putative MAPKs.

We have previously shown that OsMPK6 becomes active in response to SA [30]. Here, we monitored the activation state of OsMPK6 by examining the dual phosphorylation of MAPK at Thr and Tyr in the TEY-signature (position 225–227 in OsMPK6) [37] in calli. An immunoblot using anti-pTEpY antibody showed that a band, which is missing in *osmpk6* mutant and corresponds to that for OsMPK6 in gel mobility, was intensified by the SA-treatment in WT calli (Fig 2). The increase of the band intensity in response to SA was rather weak, which we interpret to be due to sporadically elevated basal OsMPK6 phosphorylation level because of high SA levels in rice. In the *osmpk6* mutant calli, a faster migrating band appeared, consistent with our previous observations in an in-gel kinase assay [30]. In leaves, OsMPK6 was also dually phosphorylated and another band appeared (Fig 2). These results suggest that OsMPK6 is a major MAPK activated by SA.

### Activation of OsMPK6 by OsMKK10-2 is sufficient for *WRKY45* induction and blast resistance

**Fig 3:**
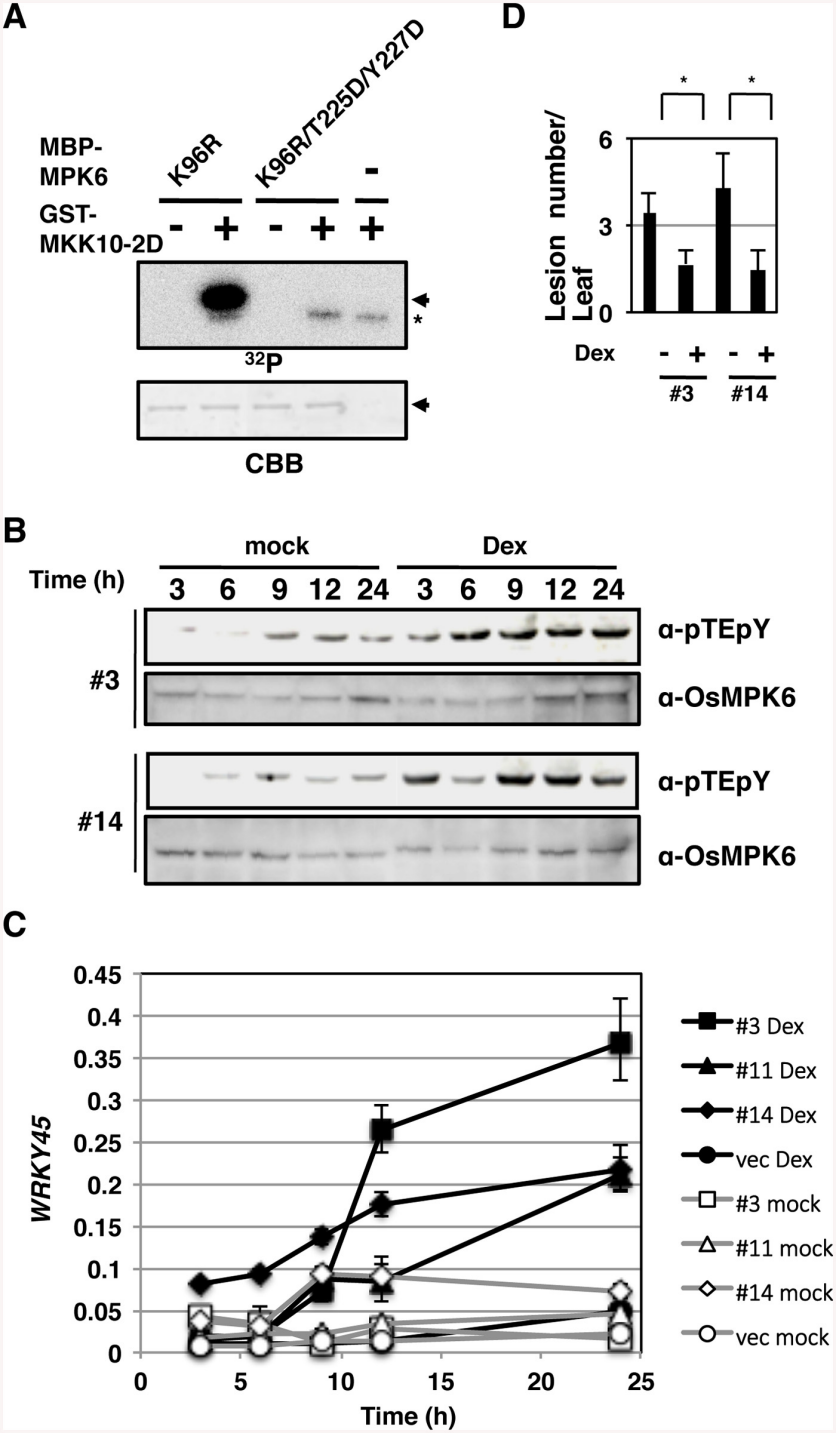
Activation of OsMPK6 is sufficient for the *WRKY45* induction and blast resistance. (A) MKK10-2D phosphorylates OsMPK6 *in vitro*. Glutathione-*S*-transferase (GST)- MKK10-2D exclusively phosphorylates Thr^225^ and Tyr^227^ of maltose binding protein (MBP)-MPK6 *in vitro*. K96R, a kinase-dead form of OsMPK6 in which Lys^96^ in the MBP-MPK6 was replaced by Arg; T225D and Y227D, OsMPK6 mutant in which Thr^225^ and Tyr^227^, respectively, were replaced by Asp; Arrows, MBP-MPK6; *, GST-MKK10-2D; CBB, Coomassie brilliant blue staining. (B) Dexamethasone (Dex)-induced MKK10-2D phosphorylates the TEY motif. Two independent *GVG-MKK10-2D* lines (#3 and #14) were treated with Dex, and proteins were detected with anti-pTEpY and anti-OsMPK6 antibodies. (C) Dex-induced *MKK10-2D* expression up-regulates *WRKY45*. *GVG*-*MKK10-2D* plants were Dex- or mock-treated, and the level of *WRKY45* transcripts relative to that of rice *ubiquitin* transcripts was determined at indicated times by RT-qPCR. (D) *MKK10-2D* expression induces blast resistance. Dex-inducible *MKK10-2D* plants were inoculated with blast fungi with or without Dex pretreatment. Values are mean lesion number/leaf (*n* = 12 biological replicates) with SEM [58]. *, *P* < 0.1 (Student’s *t*-test).

To mimic the activation of the SA pathway that leads to the activation of OsMPK6 and then WRKY45, we performed *in vitro* kinase assays using MKK10-2D and mutant forms of OsMPK6 as substrates (Figs 3A and S4). A kinase-dead form of OsMPK6, K96R, was phosphorylated by MKK10-2D *in vitro*; however, another kinase-dead form, in which, in addition to the K96R mutation, the Thr and Tyr in the TEY-signature were replaced by Asp (K96R/T225D/Y227D), was not (Fig 3A). In addition, OsMPK6 mutants in which the Tyr and Thr were independently substituted, T225D and Y227A, were less phosphorylated than the WT OsMPK6 (S4 Fig). These results indicate that MKK10-2D phosphorylates the TEY-signature of OsMPK6 specifically and suggest that OsMPK6 is activated by OsMKK10-2 through the specific phosphorylation.

Then, we expressed *MKK10-2D* in rice plants using the dexamethasone (Dex)-inducible system (GVG-*MKK10-2D*) [38] and monitored the activation of OsMPK6 by pTEpY antibody. The dual phosphorylation was induced after the Dex-treatment in two independent GVG-*MKK10-2D* lines (Fig 3B, α-pTEpY). In these plants, *WRKY45* expression (Fig 3C) and blast resistance (Fig 3D) were also induced after the Dex treatment. These results demonstrate that the activation of OsMPK6 by MKK10-2D is sufficient for the induction of *WRKY45* expression and blast resistance without exogenous SA or BTH.

OsMKK4 can also phosphorylate and activate OsMPK6 *in vitro* and *in vivo* [39] [30]. However, the induced expression of the OsMKK4 constitutively active form using the Dex induction system failed to induce *WRKY45* expression, while *phenylalanine ammonia lyase* gene, as a positive control, was induced (S5 Fig). These results suggest that OsMKK10-2, but not OsMKK4, is involved in the MAPK cascade in the SA signalling pathway leading to *WRKY45* up-regulation and blast resistance.

### OsPTP1/2 dephosphorylates a Tyr in OsMPK6 in response to ABA

**Fig 4:**
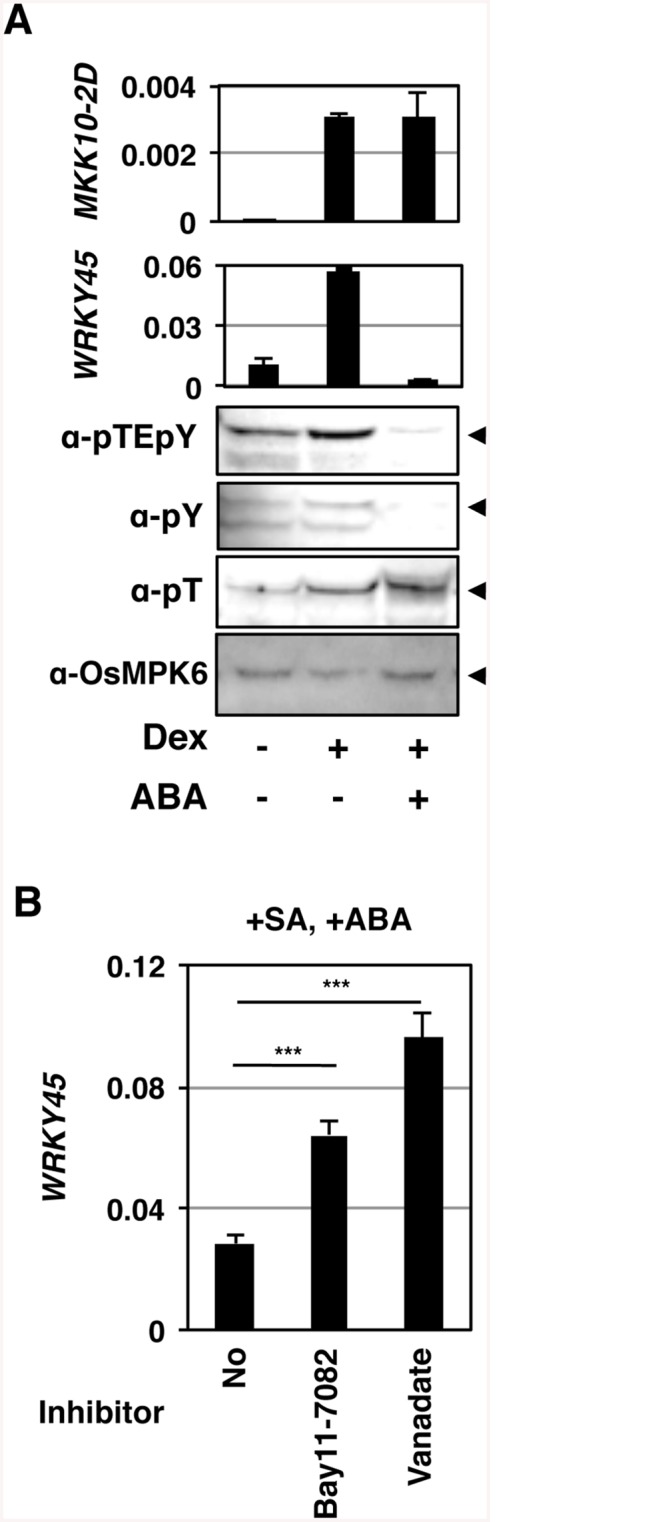
Tyr-specific dephosphorylation of OsMPK6 and suppression of *WRKY45* expression in response to ABA. (A) *GVG*-*MKK10-2D* plants (line #3) were treated with 10 μM Dex and 10 μM ABA. Means of three technical repeats (RT-qPCR relative to *ubiquitin*) with pooled samples are shown with SD. pY, anti-phosho-Tyr; pT, anti-phospho-Thr; arrowheads, OsMPK6. Similar results were obtained with another transformant line (S6 Fig). (B) Tyr-specific phosphatase (PTPase) inhibitors inhibit the suppression of salicylic acid (SA)-induced *WRKY45* transcription by ABA. WT rice plants (NB) were treated with 1 mM SA and 100 μM ABA, in the presence or absence of 50 μM Bay11-7082 or 2 mM vanadate. Means of six or more biological replicates (RT-qPCR relative to rice *ubiquitin 1*) are shown with SEM. ***, *P* < 0.001 (Student’s *t*-test).

Then, we investigated the effects of ABA on the OsMPK6–WRKY45 pathway in GVG-*MKK10-2D* lines (Fig 4A and S6 Fig). We induced OsMKK10-2D in the GVG-*MKK10-2D* transformants by Dex treatment in the presence of ABA. Interestingly, the accumulation of *WRKY45* transcripts was severely reduced by the ABA treatment, although the induction of the *MKK10-2D* transgene was unaffected by ABA (Fig 4A). These results imply a possibility that ABA-signalling interfered with the SA pathway by affecting some molecular event(s) between OsMKK10-2 activation and *WRKY45* transcription. We examined whether ABA-signalling affects the phosphorylation status of OsMPK6. Strikingly, the ABA treatment significantly lowered the dual phosphorylation level of OsMPK6 in parallel with the repression of *WRKY45* transcription (Fig 4A and S6 Fig). These results suggest that ABA-signalling dephosphorylates OsMPK6 or inhibits its phosphorylation by MKK10-2D.

**Fig 5:**
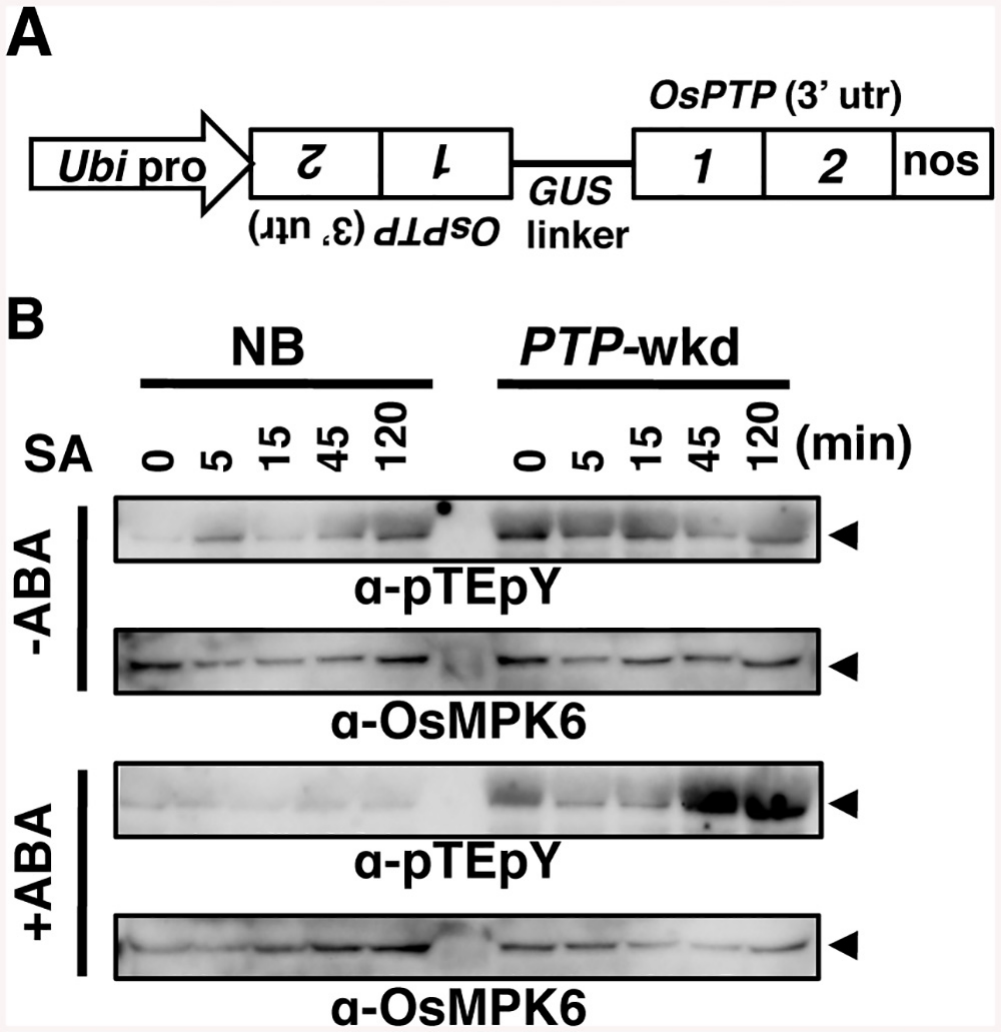
Dephosphorylation of OsMPK6 in response to ABA requires OsPTP1/2. (A) Schematic representation of the double *OsPTP1/2* knockdown (*PTP*-wkd) construct. (B) *PTP* knockdown antagonizes the TEY dephosphorylation by ABA. NB (WT) and *PTP*-wkd plants were treated for the periods indicated with 1 mM SA with or without 100 μM ABA, and proteins were detected using indicated antibodies.

In Arabidopsis, MAPK can be dephosphorylated by Ser/Thr-phosphatases, dual-specificity phosphatases, and PTPases [28] [29]. This information, taken together with our results, led us to presume that OsMPK6 could be dephosphorylated in response to ABA, rather than ABA inhibiting the MAPK kinase. To further investigate the phosphorylation state of OsMPK6, we used anti-phospho-Tyr and anti-phospho-Thr specific antibodies in an immunoblot analysis. While the phospho-Thr signal was unaffected, the phospho-Tyr signal became faint in parallel with that of dual phosphorylation (Fig 4A and S6 Fig), suggesting that the Tyr, but not Thr, of OsMPK6 was dephosphorylated in response to ABA. Based on these results, we predicted the involvement of dephosphorylation of OsMPK6 by PTPases in the action of ABA. To assess the possible involvement of PTPases, we treated rice leaf segments with SA and ABA in the presence or absence of the PTPase inhibitors, Bay11-7082 [40] and vanadate. In the absence of these inhibitors, *WRKY45* transcripts dramatically increased after the SA treatment; however, the co-treatment with ABA largely compromised the induction (S7 Fig) [25]. Meanwhile, the reduction of *WRKY45* transcript levels by ABA was significantly less in the presence of these inhibitors (Fig 4B and S7 Fig). Taken together with the results of immunodetection described above, we postulated that ABA-responsive PTPase(s) dephosphorylated/inactivated OsMPK6, which in turn deactivated WRKY45 by under-phosphorylation, leading to the reduction of *WRKY45* transcripts. The rice genome has two genes encoding putative Tyr-specific PTPases, *OsPTP1* (Os12g0174800, LOC_Os12g07590) and *OsPTP2* (Os11g0180200, LOC_Os11g07850.1) (S8 Fig), which have high homology to AtPTP1, a Tyr phosphatase that dephosphorylates MPK6 [41, 42]. Active site signature of Tyrosine phosphatase (S8 Fig), which is conserved in PTPs from all the organisms [43], is also present in OsPTP1 and -2, further lending support for these proteins being Tyr-specific phosphatases. To test this hypothesis, we generated rice transformants in which both PTPase genes were knocked down using the construct shown in Fig 5A (*PTP*-wkd). Then, we investigated the dual phosphorylation of OsMPK6 in WT [Nipponbare (NB)] and *PTP*-wkd rice plants after SA treatment in the absence or presence of ABA (Fig 5B). In NB, the dual phosphorylation level increased after the SA treatment, but the increase was completely cancelled in the presence of ABA. In *PTP*-wkd rice, ABA did not suppress the level of dual phosphorylation, which further increased after SA treatment. These results strongly suggest that OsPTP1/2 is involved in ABA-dependent dephosphorylation of OsMPK6. In the absence of ABA in *PTP*-wkd rice, the dual phosphorylation level was relatively high even without SA treatment; thus, OsPTP1/2 could also play a role in reducing the basal level of OsMPK6 phosphorylation.

**Fig 6:**
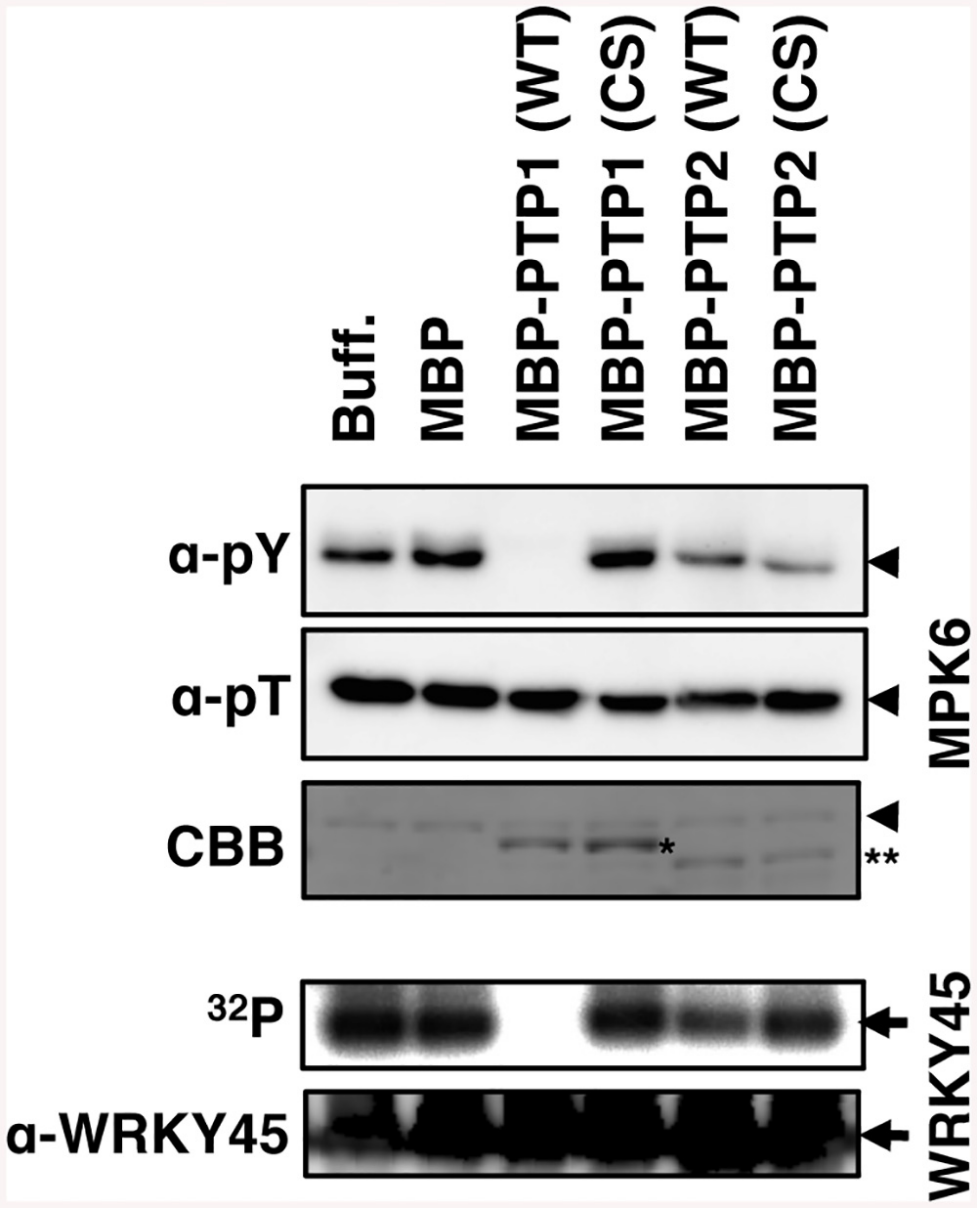
OsPTP1 directly dephosphorylates and inactivates OsMPK6 *in vitro*. To assay OsMPK6 dephosphorylating activity of the PTPs (upper panels), after incubation of WT or mutant form MBP-PTP1/2 with GST-MKK10-2D for 2 h at room temperature, phospho-Tyr and phospho-Thr of MBP-MPK6 was monitored by immuno blot using anti-pY and anti-pT antibodies, respectively. To assay WRKY45 phosphorylating activity of OsMPK6 (lower panels), after incubation of WT or mutant form of MBP-PTP1/2 with GST-MKK10-2D and unlabelled ATP for 2 h at room temperature, the mixture was further incubated with [γ-^32^P]ATP and MBP-WRKY45 for 20 min and monitored for ^32^P-incorporation into MBP-WRKY45. The lanes MBP and Buff are the controls with or without maltose binding protein, respectively. CS represents mutant PTPases with Cys-to-Ser substitutions. Coomassie brilliant blue (CBB) staining and immunoblot with anti-WRKY45 antibody are shown as loading controls. Arrowheads, MBP-MPK6; arrows, MBP-WRKY45; *, MBP-PTP1; **, MBP-PTP2.

To test whether OsPTP1/2 directly dephosphorylate OsMPK6, we performed *in vitro* dephosphorylation assays (Fig 6). Phospho-Tyr, but not phosphor-Thr, of OsMPK6, due to phosphorylation by OsMKK10-2, decreased when incubated with WT OsPTP1 fused with MBP (Fig 6, upper panels). However, the decrease of signals was not significant when incubated with a mutant OsPTP1 in which catalytically essential cysteine [44] (positions 258, S8 Fig) was replaced with serine (Fig 6). We also monitored the ability of OsMPK6 to phosphorylate WRKY45 by the addition of recombinant WRKY45 and [γ-^32^P] ATP to the system preincubated with unlabelled ATP to activate OsMPK6. The WRKY45 phosphorylation was completely abolished in the presence of WT OsPTP1, whereas the phosphorylation level remained unchanged in the presence of the mutant PTPase (Fig 6, lower panels). These results indicate that OsPTP1 directly dephosphorylates OsMPK6 and inactivates its WRKY45 phosphorylation activity. These results support the notion that OsPTP1 mediates the suppression of the SA-OsMPK6-WRKY45 pathway via ABA-signalling. We did not detect evident activities for OsPTP2 using the same reaction mixtures, possibly because of suboptimal conditions.

### Induced blast resistance was compromised by ABA or abiotic stresses

**Fig 7:**
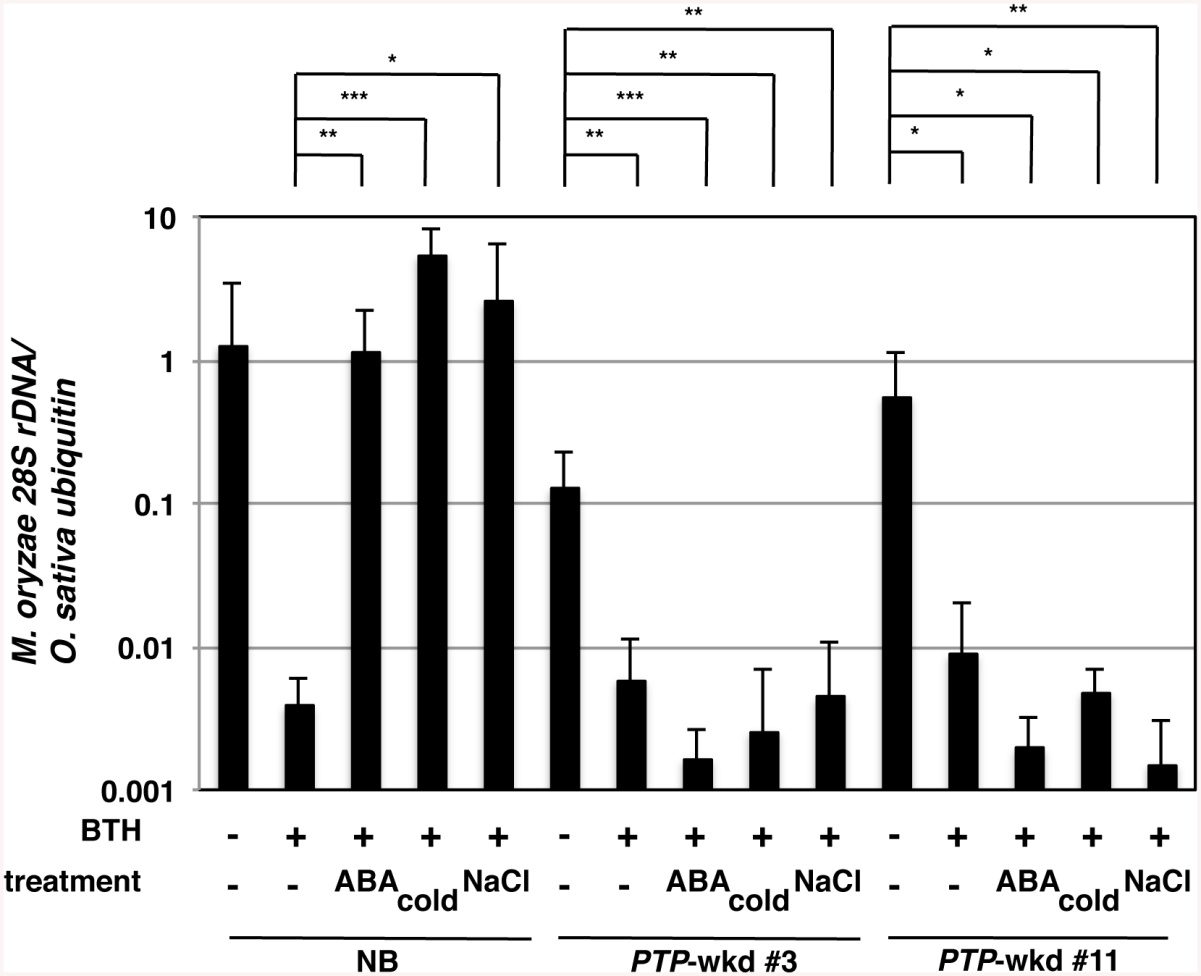
Blast resistance of WT and *PTP*-wkd plants under abiotic stress conditions. Detached leaf sheaths of wild-type Nipponbare [NB(WT)] or PTP-wkd (lines #3 and #11) plants treated with (+) or without (-) BTH (10 μM) and/or ABA (10 μM with 100 μM fluridone), or cold (15°C/9°C, day/night) were inoculated with *Magnaporthe oryzae*. Although we added fluridone with ABA to avoid feedback regulation of ABA biosynthesis, results were similar with or without fluridone. For the high salinity condition, intact plants were soaked in NaCl (250 mM) for 4 days prior to detaching leaves. Blast resistance was evaluated by quantifying *M*. *oryzae 28S rDNA* relative to that of rice *ubiquitin 1* using genomic qPCR. Means of three or more biological replicates are shown with SD. *, *P*<0.1; **, *P*<0.02; ***, *P*<0.002 (Student’s *t*-test).

BTH, a chemical defence inducer, enhances disease resistance by acting on the SA signalling pathway in various plants, including rice [45] [46]. In Arabidopsis, the effect of BTH on defence responses is compromised by high salt conditions acting through ABA signalling [24]. To test the effects of abiotic stresses on BTH-induced blast resistance in rice, we pretreated WT rice plants, NB, with 10 μM ABA, low temperature (15°C/9°C, day/night), or high salinity (250 mM NaCl) in the presence or absence of 10 μM BTH, inoculated them with *M*. *oryzae* and monitored for fungal growth (Fig 7). BTH confers a strong resistance against blast disease in rice [9]. Co-treatment with ABA compromised the resistance as reported previously [25]. Interestingly, BTH-treated plants under the cold and high salinity conditions were more highly susceptible to blast than the control plants, similar to the ABA/BTH co-treated plants (Fig 7). These results imply a possibility that the abiotic stresses mediated by ABA signalling negatively affected the BTH-induced blast resistance in rice.

### Double knockdown of *OsPTP1/2* desensitizes rice blast resistance to ABA-mediated abiotic stresses

Activation of OsMPK6 by MKK10-2D, which mimics the activation of the SA pathway by SA/BTH, conferred rice plants with blast resistance. OsPTP1/2 dephosphorylated and inactivated OsMPK6 probably in response to ABA. These results led us to examine whether *PTP*-wkd plants were less sensitive to the ABA-mediated abiotic stresses, in regard to BTH-induced disease resistance (Fig 7). Under normal conditions, BTH induced as strong a blast resistance in *PTP*-wkd plants as in NB (Fig 7). Unlike NB; however, *PTP*-wkd plants exhibited strong blast resistance even in the presence of ABA (Fig 7). Moreover, *PTP*-wkd plants showed strong blast resistance under abiotic stress conditions, low temperature (15/9°C, day/night), and high salinity (250 mM NaCl) (Fig 7). These results indicate the involvement of OsPTP1/2 in the suppression of SA/BTH-dependent blast resistance by ABA. Visible morphological and growth phenotypes were not observed in *PTP*-wkd under normal or stress conditions.

### OsPTP1/2 is involved in the ABA-dependent repression of *WRKY45* expression

**Fig 8:**
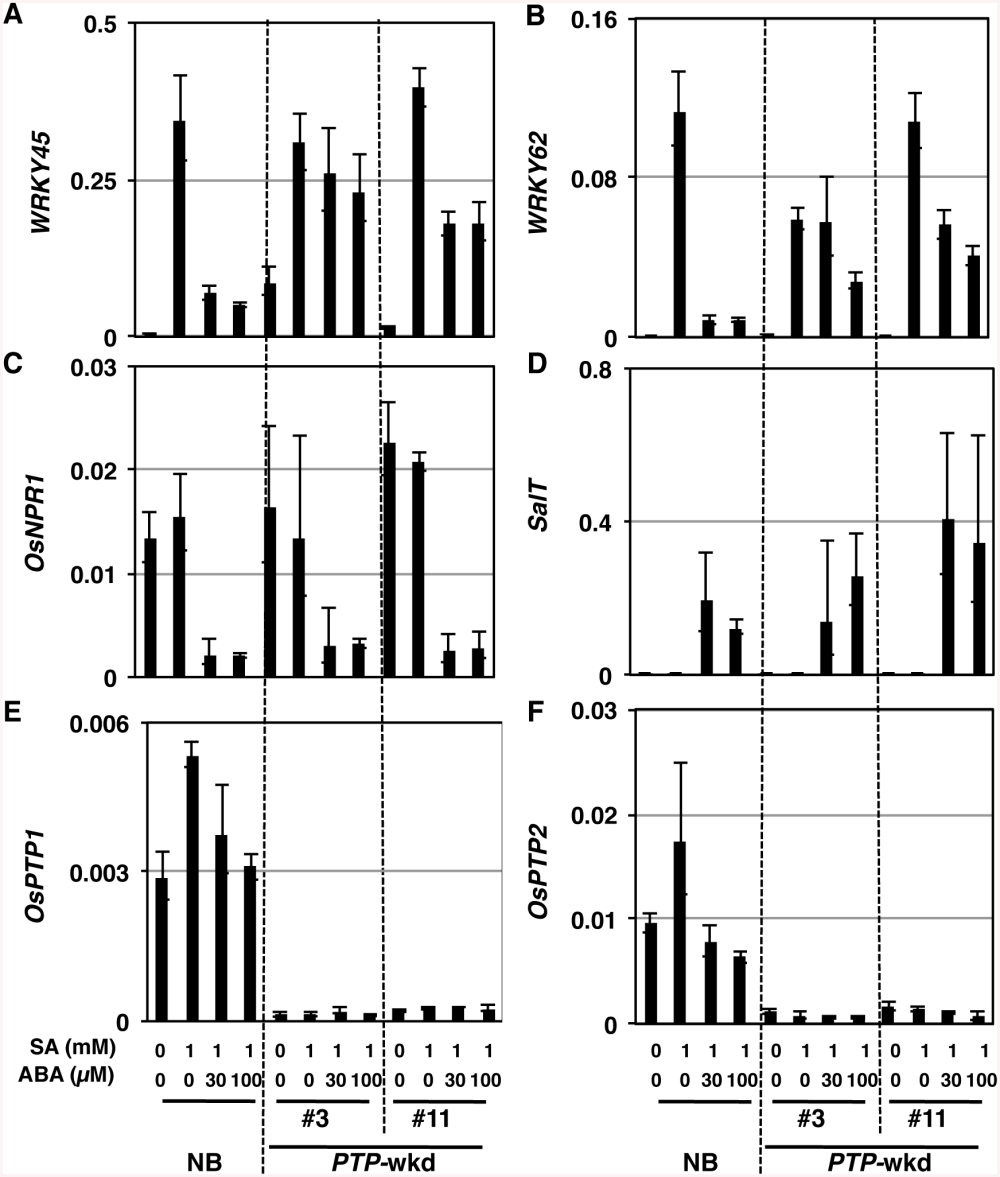
OsPTP1/2 act on the WRKY45 sub-pathway but not the OsNPR1 sub-pathway. Transcript levels of each gene (relative to that of *ubiquitin 1*) were determined by RT-qPCR using RNA extracted from leaves treated with chemicals indicated. A. *WRKY45*, B. *WRKY62*, C. *OsNPR1*, D. *SalT*, E. *OsPTP1*, F. *OsPTP2* Means of three technical repeats are shown with SD. Results of Student’s *t*-test are shown. *, *P*<0.1; **, *P*<0.05; ***, *P*<0.002; ****, *P*<0.001.

**Fig 9:**
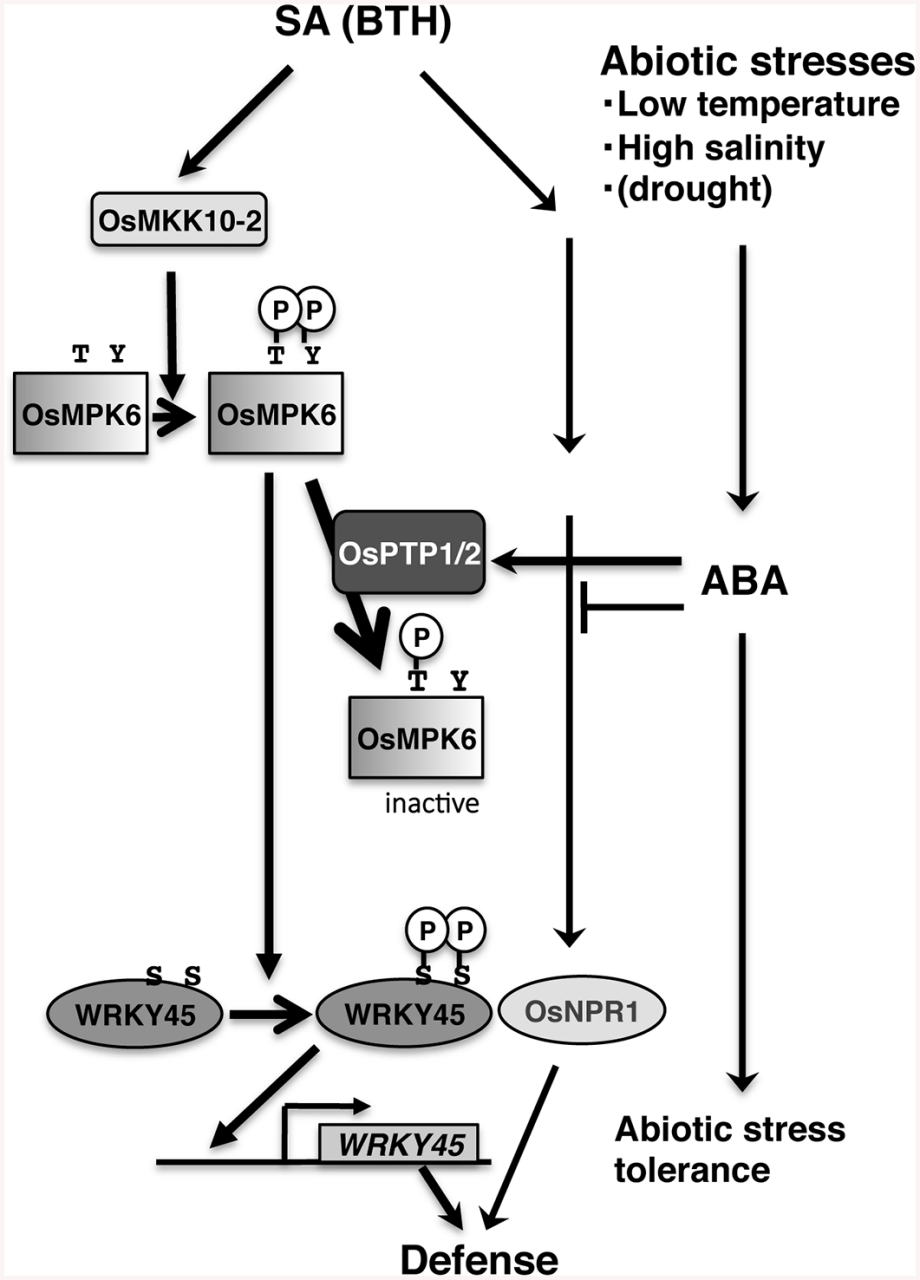
Proposed model for the mechanism underlying SA–ABA crosstalk through OsPTP1/2 against the OsMPK6–WRKY45-mediated SA pathway of the rice defence program.

SA/BTH-dependent defence system involves two independent sub-pathways, WRKY45 and OsNPR1 sub-pathways [9–12]. To investigate whether OsPTP1/2 act(s) on either sub-pathway specifically, we examined the gene expression patterns in *PTP*-wkd lines after the treatment with SA in the absence or presence of ABA (Fig 8 and S10 Fig). In the absence of ABA, *WRKY45* transcript levels in NB and *PTP*-wkd lines were within a similar range (Fig 8A). In the presence of ABA, the transcript levels were significantly decreased in NB. By contrast, the transcript levels in *PTP*-wkd plants were increased to the levels without ABA (Fig 8A). *WRKY62* is a direct target gene of WRKY45, and we have previously observed that this gene behaves similarly to *WRKY45* in response to SA/BTH [9] [14]. In this experiment (Fig 8B), the expression pattern of *WRKY62* paralleled that of *WRKY45*. We have previously reported that the expression of *OsNPR1*, as well as that of *WRKY45*, were suppressed by ABA [25]. Interestingly; however, no effect of the *OsPTP1/2* double knockdown was observed on the suppression of *OsNPR1* expression by ABA (Fig 8C). The *SalT* gene is an ABA-inducible gene [47] [25]; however, the effect of the *OsPTP1/2* double knockdown was not observed on the expression of this gene either (Fig 8D). These results suggest that OsPTP1/2 acts specifically on the WRKY45 sub-pathway of SA-signalling under abiotic stress conditions (Fig 9). The expression of *OsPTP1/2* genes was not positively affected by ABA (Fig 8E and 8F), high salinity, or low temperature condition (S9 Fig), suggesting that these genes are regulated post-transcriptionally by ABA and abiotic stresses.

## Discussion

Plants are constantly confronted to both abiotic and biotic stresses that seriously reduce their productivity. Plant responses to these stresses involve numerous complex physiological, molecular and cellular adaptations. When plants are simultaneously exposed to both abiotic and biotic stresses, plants respond in a specific manner depending on the interplays of signalling pathways that are invoked by respective stresses.

In this study, we illustrated the mechanisms underlying the WRKY45-dependent SA-signalling pathway for defence against pathogens in rice and how ABA-signalling antagonizes the WRKY45-dependent SA-signalling under abiotic stresses (Fig 9). In the absence of abiotic stresses, SA/BTH activates OsMPK6 by Thr/Tyr dual phosphorylation most probably through OsMKK10-2. The activated OsMPK6 phosphorylates one or two Ser residue(s) in the carboxyl-terminal region of WRKY45, thereby activating its transcriptional activity and conferring strong blast resistance to rice. ABA-signalling, which was activated by abiotic stresses, such as low temperature and high salinity, suppresses SA-signalling through the specific dephosphorylation of Tyr^227^ of OsMPK6 by OsPTP1/2. This results in compromised blast resistance in the presence of SA/BTH, likely because of reduced expression and activity of WRKY45.

### Different role of tyrosine protein phosphatases in Arabidopsis and rice

Tyr-specific PTPases are also encoded in dicot genomes. Of these, only Arabidopsis AtPTP1 has been functionally characterized, and the tyrosine dephosphorylating activity of AtMPK6 (MPK6) has been reported [42] [41]. Elevated levels of SA, accompanied by *PR*-gene expression, have been reported in a double mutant of the *AtPTP1* and *MKP1* phosphatase genes [41]. However, no phenotype has been found in a single mutant of the *AtPTP1* gene. Our finding for OsPTP1/2 is the first to report a function that is specific to plant PTPs; therefore, whether or not PTPs in other plant species have similar functions remains to be determined. In addition, this function is probably largely dependent on the pathosystem (combination of host and pathogen).

### Managing signalling crosstalk for further improving crops

Why have (rice) plants developed this antagonistic signalling crosstalk? Presumably, this is the mechanism behind the trade-off between the responses to biotic and abiotic stresses, which prioritizes the most life-threatening stress through the allocation of limited resources under various situations. In nature, such a system is likely to help plants to survive changing environments in a cost-efficient manner. Serious rice losses due to blast disease occurred during the cold summers of 1993 and 2003 in Japan. Other research reported that low temperatures and drought render rice plants more susceptible to blast disease [32] [33]. Moreover, high salinity conditions can breakdown the disease resistance to *Fusarium* and *Phytophthora* in tomato [48]. In our experiments, the rice plants prioritized the responses to the cold or high salinity over disease resistance, which eventually compromised the BTH-induced blast resistance. Owing to this rice response under multiple stresses, most of the rice plants died of blast disease. Considering this consequence, this trade-off mechanism does not appear to have been beneficial to plants in our experiments. Crop cultivation is often conducted under resource-rich fertile conditions, and so were our experiments. Under such conditions, the trade-off seems to be unnecessary and even harmful for plants, as well as for farmers. Therefore, down-regulating such crosstalk seems to be favourable for agriculture as long as there is no unexpected adverse side effect. Theoretically, provided that the PTPases act specifically on the SA–ABA crosstalk, there is unlikely to be any such side effects. Indeed, we have not observed any significant adverse effects on the growth of *PTP*-wkd plants so far. The same could also hold for the trade-offs between particular stress responses and/or between a particular stress and plant growth/yield. On the basis of this speculation, down-regulating a particular signalling crosstalk could be an important goal of crop breeding. So far, little is known about the molecules, besides OsPTP1/2, that directly mediate the crosstalk. To date, several signaling components have been reported to mediate signalling crosstalk; however, most of them play indirect roles in the crosstalk. Modifications of such molecules would change the balance of responses to different stresses, but they are unlikely to eliminate the crosstalk, which would maximize the defence or tolerance to both stresses [22]. Currently, only a few molecules, such as BZR1, which mediates the crosstalk between brassinosteroid- and gibberellin-signaling, as well as innate immunity [49] [50], are known to play direct roles in the signalling crosstalk. Once researchers identify more of these molecules, it should become possible to develop multi-stress-tolerant crops without penalties on yields.

## Methods

### Plant materials and growth conditions

Rice plants (*Oryza sativa* subsp. *japonica* cv. Nipponbare) were grown in a greenhouse in soil (Bonsol No. 2; Sumitomo Chemical Co., Tokyo, Japan) at 30°C/26°C (day/night) with a relative humidity (RH) of approximately 60%.

### Culturing and inoculation of *M*. *oryzae*

Culturing of the blast fungus *M*. *oryzae* (race 003) and fungal inoculations of rice plants were carried out essentially as described previously [25], with slight modifications. Briefly, *M*. *oryzae* conidia suspended in 0.02% Tween20 at a density of 150,000/ml were sprayed onto rice leaves, which had been pretreated with chemicals in 0.1 × MS in 50-ml tubes sealed with surgical tape (3M, St. Paul, MN, USA; cat# 3530–1). Detailed condition for each experiment is described below.

Normal-temperature conditions: after chemical pretreatments for 1 d in a growth chamber (30°C, 14-h day and 26°C, 10-h night; 60% RH), the solutions were removed, and the leaves were spray-inoculated with fungal conidia. The leaves were incubated in a dew chamber at 24°C for 24 h, and then for a further 5 d period in a growth chamber.

Low-temperature conditions: rice leaves were pretreated with chemicals for 2 d in a growth chamber (15°C, 14-h days and 9°C, 10-h nights; 60% RH). After fungal inoculations followed by incubation for 3 d under low-temperature conditions, solutions were removed, and leaves were further incubated for 5 d under normal-temperature conditions.

High-salinity conditions: before detaching leaves, whole plants were soaked in 250 mM NaCl for 4 d. Other procedures are the same as those of the normal condition.

Disease development was evaluated by quantifying *M*. *oryzae 28S rDNA* by qPCR [51]. Three or more biological repeats were performed for each disease resistance assay.

### Plasmid construction

Site-directed mutagenesis was performed with a QuickChange Multi Site-directed mutagenesis kit (Stratagene, La Jolla, CA, USA) according to the manufacturer’s instructions. For constructs using NanoLuc, cDNA encoding NanoLuc was subcloned from the pNL vector (Promega, Madison, WI, USA). For overexpression of WT and mutant WRKY45, cDNA amino-terminally fused with 3X myc tag was cloned into the pZH vector under the control of the maize ubiquitin promoter. For Dex-inducible *MKK10-2D*, *OsMKK10-2D* cDNA [30] was subcloned into the pINDEX vector [52]. For *PTP-wkd*, the 3′-untranslated regions of *OsPTP1* and -*2* genes were amplified using u7 (5′- CACCCGGGTATCCCTAAGGCAGGA-3′) and u8 (5′- AAATGATTCAGTTTAAACCTACTAACTCTCTTTAATTCCGT-3′), and u9 (5′- TTAGTAGGTTTAAACTGAATCATTTCTATGGAACAATCAGT-3′) and u10 (5′- AGGCCTGGGTGGGCAGGAGAAGCG-3′) primers, respectively. Then, we performed an overlapping second PCR using the u7 and u10 primers, and the products of the first reactions. Each amplified fusion gene was cloned into pENTR/D-TOPO (Invitrogen, Carlsbad, CA, USA) and subsequently transferred into the pANDA vector [53].

### Reporter assay

A reporter assay in the rice leaf sheath was essentially performed as described previously [14]. The mixture of plasmids for the expression of effectors consisted of the plasmids encoding wild-type or mutant proteins amino-terminally fused with and without NanoLuc. The effectors with or without the N-terminal NanoLuc were mixed at the ratio of 1:10. A total of 3 μg of the LUC reporter plasmid and 0.5 μg of the effecter plasmid mixture were used per assay. LUC and Nanoluc activities were determined using luciferin and furimazine as substrates, respectively, and their ratios (LUC/Nanoluc) were compared.

### Rice transformation

*Agrobacterium*-mediated transformations of rice calli were performed as described previously [54] [55]. Plants were regenerated from transformed calli by selecting for hygromycin resistance.

### Chemical treatments of leaves for protein or RNA analyses

Chemical treatments of leaves were conducted essentially as described previously [25]. Leaf blades from rice plants at the four-leaf stage were cut into segments approximately 0.5 cm long and submerged in a solution containing the chemicals prepared in 0.002% Silwet L-77. The leaf segments were incubated in the light at 30°C for the periods indicated. We applied ABA 1 h before SA or Dex treatments. The PTPase inhibitors were applied 1 h before the ABA treatment.

### Immunoblot assays

Immunoblot assays were carried out essentially as described previously [15]. Proteins were extracted with 50 mM Hepes-KOH, pH 7.5, containing Complete Protease Inhibitor Cocktail (Roche Diagnostics, Mannheim, Germany), 1 mM PMSF, and protein phosphatase inhibitor cocktails (phosphatase inhibitor cocktail 1 and 2; Sigma, St Louis, MO, USA) or alternatively PhosTop (Roche Diagnostics). For the treatment with PPase, the protein phosphatase inhibitor cocktails were excluded. After centrifugation, supernatants (containing 6–20 μg protein) were subjected to sodium dodecyl sulfate polyacrylamide gel electrophoresis (SDS-PAGE; 10% 29:1acrylamide:bis-acrylamide), followed by electroblotting. In the case of the Phos-Tag SDS-PAGE, 10 μM Phos-Tag acrylamide (Wako, Tokyo, Japan) and 20 μM Zn(NO_3_)_2_ were included in the 7.5% polyacrylamide gel (29:1 acrylamide:bis-acrylamide). Other procedures were performed as described previously [36].

Immunodetection was performed using SNAPid (Millipore, Billerica, MA, USA) according to the manufacturer’s instructions. The antibodies used were as follows: anti-pTEpY (Promega, cat# V8031) at a 1/1,500 dilution; anti-OsMPK6 [39] at a 1/1,500 dilution; anti-phosphoenolpyruvate carboxylase [56] at a 1/30,000 dilution; anti-pY (Millipore, clone 4G10 platinum) at a 1/1,500 dilution; anti-pT [Promega, anti-pT183 MAPK pAb (rabbit)] at a 1/4,000 dilution; and anti-WRKY45 [15] at a 1/300 dilution.

### RT-qPCR

Total RNA was isolated from rice leaves treated with chemicals as described above using Trizol reagent (Invitrogen). cDNA was synthesized using ReverTraAce (Toyobo, Tokyo, Japan). Quantitative PCR was run on a Thermal Cycler Dice TP800 system (Takara Bio, Tokyo, Japan) using the SYBR premix ExTaq mixture (Takara Bio) as described previously [9]. Sequences of primers used for RT-qPCR are listed in S1 Table.

### Phosphorylation and dephosphorylation assay *in vitro*

Phosphorylation and dephosphorylation assays were carried out as described previously [57] [39], with modifications. GST-MKK10-2D and MBP-MPK6 (WT or mutant) were incubated in reaction buffer (10 mM Hepes-KOH, pH 7.5, 5 mM EGTA, 20 mM MgCl_2_, 1 mM DTT) containing 0.5 mM ATP at 25°C for 20 min. For dephosphorylation assays, MBP-PTP1 or -2 was added and the reaction mixture was incubated for an additional 20 min. For WRKY45 phosphorylation activity assays, the same reaction mixtures were pre-incubated for 20 min, and reactions were initiated by adding MBP-WRKY45 and 37 kBq [γ-^32^P]ATP. The mixtures were incubated for an additional 20 min, and then terminated by adding Laemmle’s sample buffer and boiling. Labelled proteins were analysed by SDS-PAGE. Coomassie brilliant blue staining was performed as loading controls.

### Accession numbers

WRKY45: Os05g0322900, LOC_Os05g25770

OsMKK10-2: Os03g0225100, LOC_Os03g12390.1

OsMPK6: Os06g0154500, LOC_Os06g06090

OsPTP1: Os12g0174800, LOC_Os12g07590

OsPTP2: Os11g0180200, LOC_Os11g07850.1

## Supporting Information

S1 Table Primer sequences used for qPCR. (XLSX)

S1 Fig Mapping of the phosphorylated region of WRKY45. (A) Schematic representation of partial WRKY45 (W45) polypeptides. Numbers above the boxes indicate the numbers of Ser or Thr residues in the sub-regions. (B) Coomassie brilliant blue (CBB)-staining of the partial WRKY45 polypeptides fused with maltose binding proteins (MBPs). MBP-lacZa, negative control protein expressed from empty vector. (C) Phosphorylation *in vitro* of the partial WRKY45 polypeptides by OsMPK6 shown by ^32^P incorporation from [γ-^32^P]ATP.
(PPTX)

S2 Fig Determination of phosphorylation sites by Ala-substitution of WRKY45 sub-fragments. Subfragments of WRKY45 (239–292 and 281–326) polypeptides fused with MBPs were assayed for phosphorylation *in vitro*. Thr^266^ and Ser^269^ in WRKY45 (239–292) and Ser^294^, Ser^295^, and Ser^299^ in WRKY45 (281–326) were substituted with Ala in each mutant polypeptide and incubated with OsMPK6 as described in Materials and Methods. An arrowhead indicates phosphorylated WRKY45 polypeptides. Phosphorylated amino acids deduced from the results are underlined. CBB staining of the gel is shown with a schematic representation of phosphorylation sites below.
(PPTX)

S3 Fig Phos-Tag SDS-PAGE of WRKY45 proteins. The same extracts as those in Fig 2E were separated on Phos-Tag SDS-PAGE. Proteins were detected by immunoblot assay with anti-myc antibody.
(PPTX)

S4 Fig GST-MKK10-2D phosphoylates WT and mutant MBP-MPK6 protein *in vitro*. Phosphorylation of WT and mutant forms (Y227D and T225A) of MBP-MPK6 proteins were assayed using [γ-^32^P]ATP as a substrate as described in Materials and Methods, CBB, loading control.
(PPTX)

S5 Fig Gene expression in GVG*-MKK4DD* transformant cells. Transcript levels of *WRKY45* and *phenylalanine ammonia-lyase* (*PAL*) genes (relative to that of *ubiquitin 1*) were determined by qRT-PCR. The same results with different scale are shown for *WRKY45* in an inset.
(PPTX)

S6 Fig Tyr dephosphorylation of OsMPK6 by OsPTP1/2 in response to ABA. GVG*-MKK10-2D* line #14 was analyzed as in Fig 5A.
(PPTX)

S7 Fig PTPase inhibitors inhibit the suppression of SA-induced *WRKY45* transcription by ABA. WT rice plants were treated with 1 mM SA and increasing concentrations of ABA, in the presence or absence of 2 mM vanadate or 50 μM Bay11-7082. *, *P*<0.05; **, *P*<0.001 (Student’s *t*-test).
(PPTX)

S8 Fig Alignment of amino acid sequences for OsPTP1, OsPTP2, and AtPTP1 proteins. Conserved residues are boxed. *, catalytically essential Cys residue. Active site signature [(I/V)HCXAGXXR(S/T)G] conserved in PTPs from all organisms is underlined.
(PPTX)

S9 Fig Responses of *OsPTP1* and *-2* transcripts to high salinity and low temperature. Transcript levels of *OsPTP1* and *-2* (relative to that of rice *ubiquitin 1*) in NB (WT) plants treated with 250 mM NaCl or 8°C for indicated day length were determined by RT-qPCR.
(PPTX)

S10 Fig OsPTP1/2 act on the WRKY45 sub-pathway but not the OsNPR1 sub-pathway. Transcript levels of *WRKY45* and *OsNPR1* (relative to that of rice *ubiquitin 1*) in NB (WT) and *PTP*-wkd plants treated with 1 mM SA in the absence or presence of ABA (30 μM) were determined by RT-qPCR. Means of eight biological replicates are shown with SEM. ***, *P*<0.002 (Student’s *t*-test).
(PPTX)

## References

[1] Matyssek R, Agerer R, Ernst D, Munch JC, Osswald W, Pretzsch H, et al The plant's capacity in regulating resource demand. Plant biology. 2005;7(6):560–80. 10.1055/s-2005-872981 . 16388460

[2] Tian D, Traw MB, Chen JQ, Kreitman M, Bergelson J. Fitness costs of R-gene-mediated resistance in Arabidopsis thaliana. Nature. 2003;423(6935):74–7. 10.1038/nature01588 . 12721627

[3] Fujita M, Fujita Y, Noutoshi Y, Takahashi F, Narusaka Y, Yamaguchi-Shinozaki K, et al Crosstalk between abiotic and biotic stress responses: a current view from the points of convergence in the stress signaling networks. Curr Opin Plant Biol. 2006;9(4):436–42. 10.1016/j.pbi.2006.05.014 . 16759898

[4] Pieterse CM, Van der Does D, Zamioudis C, Leon-Reyes A, Van Wees SC. Hormonal modulation of plant immunity. Annu Rev Cell Dev Biol. 2012;28:489–521. 10.1146/annurev-cellbio-092910-154055 . 22559264

[5] Sharma R, De Vleesschauwer D, Sharma MK, Ronald PC. Recent advances in dissecting stress-regulatory crosstalk in rice. Mol Plant. 2013;6(2):250–60. 10.1093/mp/sss147 . 23292878

[6] Vert G, Chory J. Crosstalk in cellular signaling: background noise or the real thing? Dev Cell. 2011;21(6):985–91. 10.1016/j.devcel.2011.11.006 22172668 PMC3281494

[7] DenancÈ N, S·nchez-Vallet A, Goffner D, Molina A. Disease resistance or growth: the role of plant hormones in balancing immune responses and fitness costs. Frontiers in Plant Science. 2013;4 10.3389/fpls.2013.00155 PMC3662895 23745126

[8] Cao H, Glazebrook J, Clarke JD, Volko S, Dong X. The Arabidopsis NPR1 gene that controls systemic acquired resistance encodes a novel protein containing ankyrin repeats. Cell. 1997;88(1):57–63. . 9019406 10.1016/s0092-8674(00)81858-9

[9] Shimono M, Sugano S, Nakayama A, Jiang CJ, Ono K, Toki S, et al Rice WRKY45 plays a crucial role in benzothiadiazole-inducible blast resistance. Plant Cell. 2007;19(6):2064–76. 10.1105/tpc.106.046250 17601827 PMC1955718

[10] Sugano S, Jiang C-J, Miyazawa S-I, Masumoto C, Yazawa K, Hayashi N, et al Role of OsNPR1 in rice defense program as revealed by genome-wide expression analysis. Plant Molecular Biology. 2010;74(6):549–62. Epub 2010/10/07. 10.1007/s11103-010-9695-3 . 20924648

[11] Takatsuji H, Jiang C-J, Sugano S. Salicylic acid signaling pathway in rice and the potential applications of its regulators. JARQ. 2010;44(3):217–23. ISI:000284669300001.

[12] Nakayama A, Fukushima S, Goto S, Matsushita A, Shimono M, Sugano S, et al Genome-wide identification of WRKY45-regulated genes that mediate benzothiadiazole-induced defense responses in rice. BMC Plant Biology. 2013;13(1):150 10.1186/1471-2229-13-150 24093634 PMC3850545

[13] Shimono M, Koga H, Akagi AYA, Hayashi N, Goto S, Sawada M, et al Rice WRKY45 plays important roles in fungal and bacterial disease resistance. Molecular Plant Pathology. 2012;13(1):83–94. 10.1111/j.1364-3703.2011.00732.x 21726399 PMC6638719

[14] Inoue H, Hayashi N, Matsushita A, Xinqiong L, Nakayama A, Sugano S, et al Blast resistance of CC-NB-LRR protein Pb1 is mediated by WRKY45 through protein-protein interaction. Proc Natl Acad Sci U S A. 2013;110(23):9577–82. 10.1073/pnas.1222155110 23696671 PMC3677490

[15] Matsushita A, Inoue H, Goto S, Nakayama A, Sugano S, Hayashi N, et al Nuclear ubiquitin proteasome degradation affects WRKY45 function in the rice defense program. The Plant Journal. 2013;73(2):302–13. 10.1111/tpj.12035 23013464 PMC3558880

[16] Vlot AC, Dempsey DA, Klessig DF. Salicylic Acid, a multifaceted hormone to combat disease. Annu Rev Phytopathol. 2009;47:177–206. 10.1146/annurev.phyto.050908.135202 . 19400653

[17] Cutler SR, Rodriguez PL, Finkelstein RR, Abrams SR. Abscisic acid: emergence of a core signaling network. Annu Rev Plant Biol. 2010;61:651–79. 10.1146/annurev-arplant-042809-112122 . 20192755

[18] Mauch-Mani B, Mauch F. The role of abscisic acid in plant-pathogen interactions. Curr Opin Plant Biol. 2005;8(4):409–14. 10.1016/j.pbi.2005.05.015 . 15939661

[19] Asselbergh B, De Vleesschauwer D, Hofte M. Global switches and fine-tuning-ABA modulates plant pathogen defense. Mol Plant Microbe Interact. 2008;21(6):709–19. 10.1094/MPMI-21-6-0709 . 18624635

[20] Cao FY, Yoshioka K, Desveaux D. The roles of ABA in plant-pathogen interactions. J Plant Res. 2011;124(4):489–99. 10.1007/s10265-011-0409-y . 21380629

[21] De Vleesschauwer D, Gheysen G, Höfte M. Hormone defense networking in rice: tales from a different world. Trends in Plant Science. 2013;18(10):555–65. 10.1016/j.tplants.2013.07.002 . 23910453

[22] Takatsuji H, Jiang C-J. Plant hormone crosstalks under biotic stresses In: Tran L-SP, Pal S, editors. Phytohormones: a window to metabolism, signaling and biotechnological applications: Springer New York; 2014 p. 323–50.

[23] Audenaert K, De Meyer GB, Hofte MM. Abscisic acid determines basal susceptibility of tomato to Botrytis cinerea and suppresses salicylic acid-dependent signaling mechanisms. Plant Physiol. 2002;128(2):491–501. 10.1104/pp.010605 11842153 PMC148912

[24] Yasuda M, Ishikawa A, Jikumaru Y, Seki M, Umezawa T, Asami T, et al Antagonistic interaction between systemic acquired resistance and the abscisic acid-mediated abiotic stress response in Arabidopsis. Plant Cell. 2008;20(6):1678–92. Epub 2008/07/01. tpc.107.054296 [pii] 10.1105/tpc.107.054296 . 18586869 PMC2483369

[25] Jiang C-J, Shimono M, Sugano S, Kojima M, Yazawa K, Yoshida R, et al Abscisic acid interacts antagonistically with salicylic acid signaling pathway in rice–Magnaporthe grisea Interaction. Molecular Plant-Microbe Interactions. 2010;23(6):791–8. 10.1094/MPMI-23-6-0791 20459318

[26] Mao G, Meng X, Liu Y, Zheng Z, Chen Z, Zhang S. Phosphorylation of a WRKY transcription factor by two pathogen-responsive MAPKs drives phytoalexin biosynthesis in Arabidopsis. Plant Cell. 2011;23(4):1639–53. Epub 2011/04/19. 10.1105/tpc.111.084996 21498677 PMC3101563

[27] Ishihama N, Yamada R, Yoshioka M, Katou S, Yoshioka H. Phosphorylation of the Nicotiana benthamiana WRKY8 Transcription Factor by MAPK Functions in the Defense Response. Plant Cell. 2011 Epub 2011/03/10. tpc.110.081794 [pii] 10.1105/tpc.110.081794 . 21386030 PMC3082260

[28] Bartels S, Gonzalez Besteiro MA, Lang D, Ulm R. Emerging functions for plant MAP kinase phosphatases. Trends Plant Sci. 2010;15(6):322–9. Epub 2010/05/11. 10.1016/j.tplants.2010.04.003 . 20452268

[29] Rodriguez MC, Petersen M, Mundy J. Mitogen-activated protein kinase signaling in plants. Annu Rev Plant Biol. 2010;61:621–49. 10.1146/annurev-arplant-042809-112252 . 20441529

[30] Ueno Y, Yoshida R, Kishi-Kaboshi M, Matsushita A, Jiang C-J, Goto S, et al MAP kinases phosphorylate rice WRKY45. Plant Signaling & Behavior. 2013;8(6):e24510. 23603961 10.4161/psb.24510 PMC3908937

[31] Kahn RP, Libby JL. The effect of environmental factors and plant ages on the infection of rice by the blast fungus, *Pyricularia oryzae* . Phytopathology. 1958;48:25–30.

[32] Bonman JM, Sanchez LM, Mackill AO. Effects of water deficit on rice blast. II. Disease-development in the field. J Plant Prot Trop. 1988;5:67–73.

[33] Gill MA, Bonman JM. Effects of water deficit on rice blast. I. Influence of water deficit on components of resistance. J Plant Prot Trop. 1988;5:61–6.

[34] Koga H, Dohi K, Mori M. Abscisic acid and low temperatures suppress the whole plant-specific resistance reaction of rice plants to the infection of Magnaporthe grisea. Physiological and Molecular Plant Pathology. 2004;65(1):3–9. 10.1016/j.pmpp.2004.11.002

[35] Yazawa K, Jiang C-J, Kojima M, Sakakibara H, Takatsuji H. Reduction of abscisic acid levels or inhibition of abscisic acid signaling in rice during the early phase of Magnaporthe oryzae infection decreases its susceptibility to the fungus. Physiological and Molecular Plant Pathology. 2012;78:1–7. 10.1016/j.pmpp.2011.12.003

[36] Kinoshita E, Kinoshita-Kikuta E. Improved Phos-tag SDS-PAGE under neutral pH conditions for advanced protein phosphorylation profiling. Proteomics. 2011;11(2):319–23. 10.1002/pmic.201000472 . 21204258

[37] Anderson NG, Maller JL, Tonks NK, Sturgill TW. Requirement for integration of signals from two distinct phosphorylation pathways for activation of MAP kinase. Nature. 1990;343(6259):651–3. 2154696 10.1038/343651a0

[38] Aoyama T, Chua NH. A glucocorticoid-mediated transcriptional induction system in transgenic plants. Plant J. 1997;11(3):605–12. Epub 1997/03/01. . 9107046 10.1046/j.1365-313x.1997.11030605.x

[39] Kishi-Kaboshi M, Okada K, Kurimoto L, Murakami S, Umezawa T, Shibuya N, et al A rice fungal MAMP-responsive MAPK cascade regulates metabolic flow to antimicrobial metabolite synthesis. Plant J. 2010;63(4):599–612. 10.1111/j.1365-313X.2010.04264.x 20525005 PMC2988419

[40] Krishnan N, Bencze G, Cohen P, Tonks NK. The anti-inflammatory compound BAY 11–7082 is a potent inhibitor of Protein Tyrosine Phosphatases. FEBS J. 2013 10.1111/febs.12283 . 23578302 PMC3712534

[41] Bartels S, Anderson JC, Gonzalez Besteiro MA, Carreri A, Hirt H, Buchala A, et al MAP kinase phosphatase1 and protein tyrosine phosphatase1 are repressors of salicylic acid synthesis and SNC1-mediated responses in Arabidopsis. Plant Cell. 2009;21(9):2884–97. 10.1105/tpc.109.067678 19789277 PMC2768924

[42] Gupta R. Redox control of protein tyrosine phosphatases and mitogen-activated protein kinases in plants. Plant Physiology. 2003;132(3):1149–52. 10.1104/pp.103.020792 12857797 PMC1540326

[43] Guan KL. The mitogen activated protein kinase signal transduction pathway: from cell surface to the nucleus. Cell Signal. 1994;6:581–9. 7857762 10.1016/0898-6568(94)90041-8

[44] Xu Q, Fu HH, Gupta R, Luan S. Molecular characterization of a tyrosine-specific protein phosphatase encoded by a stress-responsive gene in Arabidopsis. The Plant Cell. 1998;10(5):849–57. 9596642 10.1105/tpc.10.5.849 PMC144019

[45] Görlach J, Volrath S, Knauf-Beiter G, Hengy G, Beckhove U, Kogel KH, et al Benzothiadiazole, a novel class of inducers of systemic acquired resistance, activates gene expression and disease resistance in wheat. Plant Cell. 1996;8(4):629–43. 10.1105/tpc.8.4.629 8624439 PMC161125

[46] Lawton KA, Friedrich L, Hunt M, Weymann K, Delaney T, Kessmann H, et al Benzothiadiazole induces disease resistance in Arabidopsis by activation of the systemic acquired resistance signal transduction pathway. The Plant Journal. 1996;10(1):71–82. 10.1046/j.1365-313X.1996.10010071.x 8758979

[47] Rabbani MA, Maruyama K, Abe H, Khan MA, Katsura K, Ito Y, et al Monitoring expression profiles of rice genes under cold, drought, and high-salinity stresses and abscisic acid application using cDNA microarray and RNA gel-blot analyses. Plant Physiol. 2003;133(4):1755–67. 10.1104/pp.103.025742 14645724 PMC300730

[48] Besri M. Effects of salinity on plant diseases development In: Lieth H, Al Masoom A, editors. Towards the rational use of high salinity tolerant plants. Tasks for vegetation science. 28: Springer Netherlands; 1993 p. 67–74.

[49] Li QF, Wang C, Jiang L, Li S, Sun SS, He JX. An interaction between BZR1 and DELLAs mediates direct signaling crosstalk between brassinosteroids and gibberellins in Arabidopsis. Sci Signal. 2012;5(244):ra72 10.1126/scisignal.2002908 . 23033541

[50] Lozano-Duran R, Macho AP, Boutrot F, Segonzac C, Somssich IE, Zipfel C. The transcriptional regulator BZR1 mediates trade-off between plant innate immunity and growth. Elife. 2013;2:e00983 10.7554/eLife.00983 24381244 PMC3875382

[51] Qi M, Yang Y. Quantification of magnaporthe grisea during infection of rice plants using real-time polymerase chain reaction and northern blot/phosphoimaging analyses. Phytopathology. 2002;92(8):870–6. 10.1094/PHYTO.2002.92.8.870 18942966

[52] Ouwerkerk P, de Kam R, Hoge J, Meijer A. Glucocorticoid-inducible gene expression in rice. Planta. 2001;213:370–8. 10.1007/s004250100583 11506359

[53] Miki D, Shimamoto K. Simple RNAi vectors for stable and transient suppression of gene function in rice. Plant and Cell Physiology. 2004;45(4):490–5. 10.1093/pcp/pch048 15111724

[54] Hiei Y, Ohta S, Komari T, Kumashiro T. Efficient transformation of rice (Oryza sativa L.) mediated by Agrobacterium and sequence analysis of the boundaries of the T-DNA. The Plant Journal. 1994;6(2):271–82. 10.1046/j.1365-313X.1994.6020271.x 7920717

[55] Fuse T, Sasaki T, Yano M. Ti-plasmid vectors useful for functional analysis of rice genes. Plant BioTechology. 2001;18:219–22.

[56] Ueno Y, Imanari E, Emura J, Yoshizawa-Kumagaye K, Nakajima K, Inami K, et al Immunological analysis of the phosphorylation state of maize C4-form phosphoenolpyruvate carboxylase with specific antibodies raised against a synthetic phosphorylated peptide. Plant J. 2000;21(1):17–26. . 10652147 10.1046/j.1365-313x.2000.00649.x

[57] Huang Y, Li H, Gupta R, Morris PC, Luan S, Kieber JJ. ATMPK4, an Arabidopsis homolog of mitogen-activated protein kinase, is activated in vitro by AtMEK1 through threonine phosphorylation. Plant Physiology. 2000;122(4):1301–10. 10.1104/pp.122.4.1301 10759527 PMC58966

[58] Akagi A, Jiang C-J, Takatsuji H. Magnaporthe oryzae Inoculation of rice seedlings by spraying with a spore suspension. Bioprotocol. 2015;5(11):e1486.

