# Supplementary material for: Abiotic Stresses Antagonize the Rice Defence Pathway through the Tyrosine-Dephosphorylation of OsMPK6
Source: PLoS Pathog. 2015 Oct 20;11(10):e1005231. doi: 10.1371/journal.ppat.1005231 (PMC4617645; doi:10.1371/journal.ppat.1005231)
Supplement: S1 Fig — (A) Schematic representation of partial WRKY45 (W45) polypeptides. Numbers above the boxes indicate the numbers of Ser or Thr residues in the sub-regions. (B) Coomassie brilliant blue (CBB)-staining of the partial WRKY45 polypeptides fused with maltose binding proteins (MBPs). MBP-lacZa, negative control protein expressed from empty vector. (C) Phosphorylation in vitro of the partial WRKY45 polypeptides by OsMPK6 shown by 32P incorporation from [γ-32P]ATP. (PPTX) [file ppat.1005231.s002.pptx]

## Slide 1
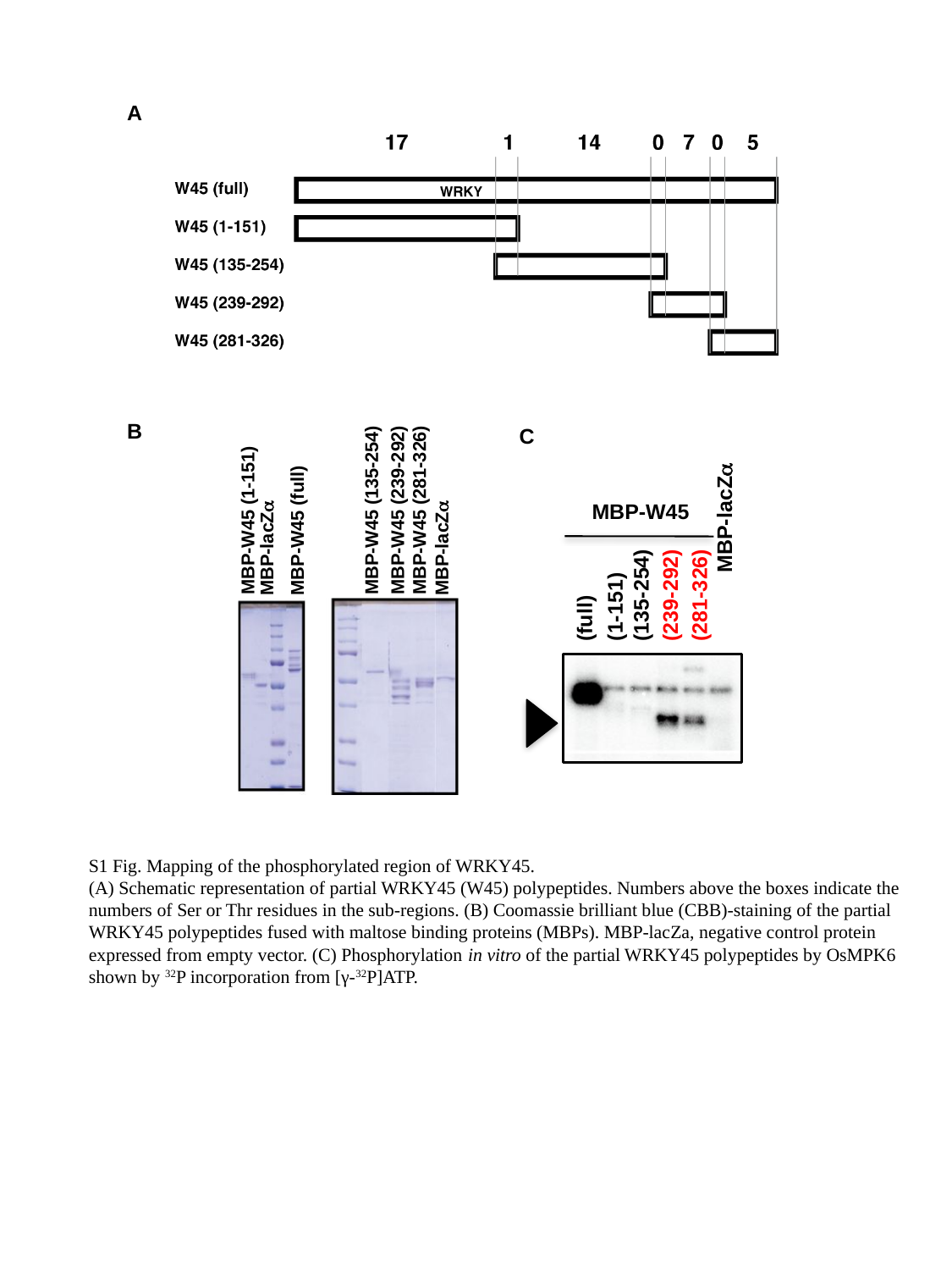

A
B
C
MBP-W45
MBP-lacZa
MBP-W45 (135-254)
MBP-W45 (239-292)
MBP-W45 (281-326)
MBP-W45 (1-151)
MBP-W45 (full)
MBP-lacZa
MBP-lacZa
(135-254)
(239-292)
(281-326)
(1-151)
(full)
S1 Fig. Mapping of the phosphorylated region of WRKY45.
(A) Schematic representation of partial WRKY45 (W45) polypeptides. Numbers above the boxes indicate the numbers of Ser or Thr residues in the sub-regions. (B) Coomassie brilliant blue (CBB)-staining of the partial WRKY45 polypeptides fused with maltose binding proteins (MBPs). MBP-lacZa, negative control protein expressed from empty vector. (C) Phosphorylation in vitro of the partial WRKY45 polypeptides by OsMPK6 shown by 32P incorporation from [γ-32P]ATP.
