# Supplementary material for: Abiotic Stresses Antagonize the Rice Defence Pathway through the Tyrosine-Dephosphorylation of OsMPK6
Source: PLoS Pathog. 2015 Oct 20;11(10):e1005231. doi: 10.1371/journal.ppat.1005231 (PMC4617645; doi:10.1371/journal.ppat.1005231)
Supplement: S2 Fig — Subfragments of WRKY45 (239–292 and 281–326) polypeptides fused with MBPs were assayed for phosphorylation in vitro. Thr266 and Ser269 in WRKY45 (239–292) and Ser294, Ser295, and Ser299 in WRKY45 (281–326) were substituted with Ala in each mutant polypeptide and incubated with OsMPK6 as described in Materials and Methods. An arrowhead indicates phosphorylated WRKY45 polypeptides. Phosphorylated amino acids deduced from the results are underlined. CBB staining of the gel is shown with a schematic representation of phosphorylation sites below. (PPTX) [file ppat.1005231.s003.pptx]

## Slide 1
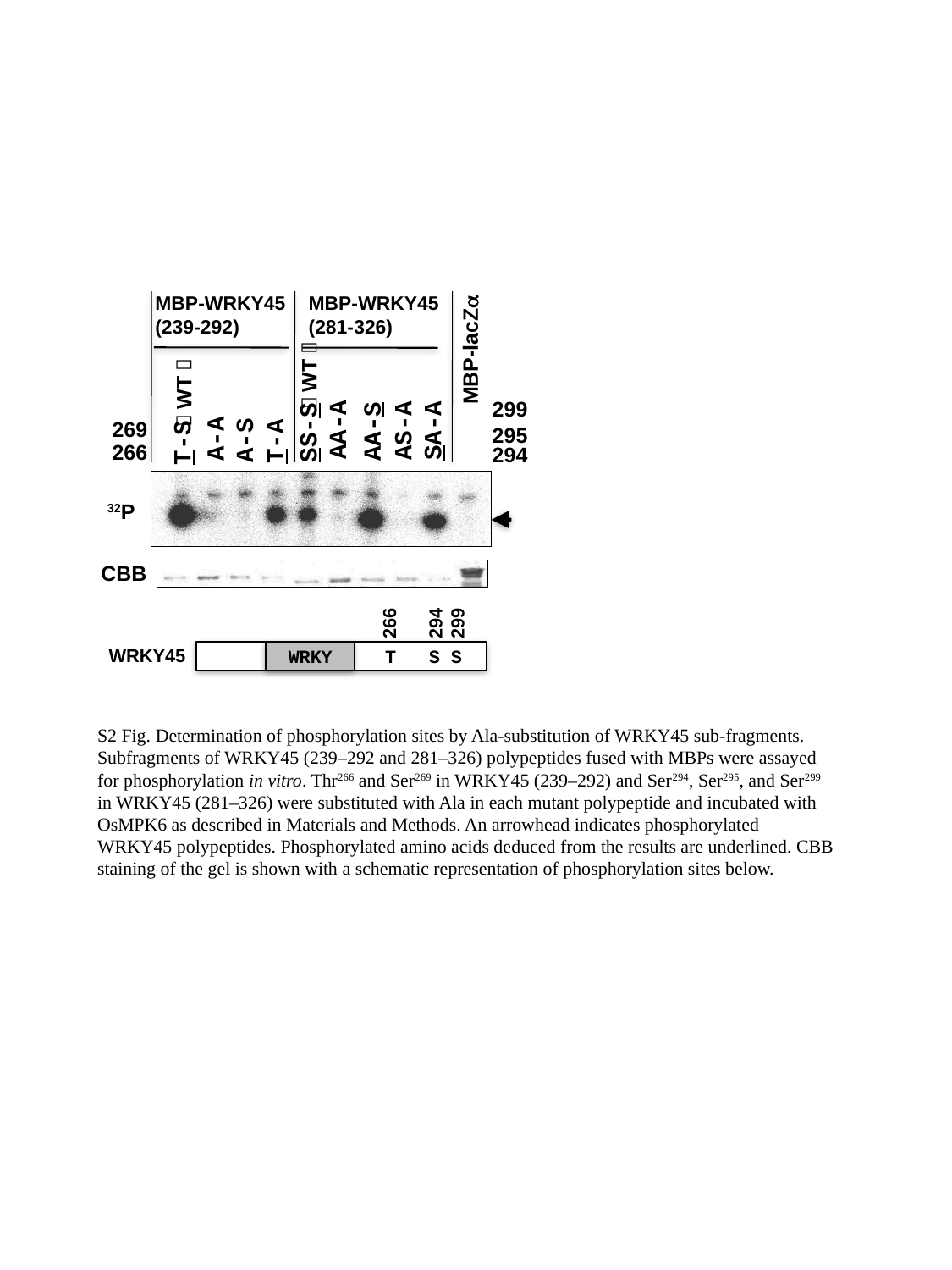

MBP-WRKY45
(239-292)
MBP-WRKY45
(281-326)
MBP-lacZa
（WT）
（WT）
299
269
AA-A
295
AS-A
SA-A
AA-S
SS-S
A-A
A-S
T-A
T-S
266
294
32P
CBB
266
294
299
WRKY
T S S
WRKY45
S2 Fig. Determination of phosphorylation sites by Ala-substitution of WRKY45 sub-fragments.
Subfragments of WRKY45 (239–292 and 281–326) polypeptides fused with MBPs were assayed for phosphorylation in vitro. Thr266 and Ser269 in WRKY45 (239–292) and Ser294, Ser295, and Ser299 in WRKY45 (281–326) were substituted with Ala in each mutant polypeptide and incubated with OsMPK6 as described in Materials and Methods. An arrowhead indicates phosphorylated WRKY45 polypeptides. Phosphorylated amino acids deduced from the results are underlined. CBB staining of the gel is shown with a schematic representation of phosphorylation sites below.
