# Supplementary material for: Abiotic Stresses Antagonize the Rice Defence Pathway through the Tyrosine-Dephosphorylation of OsMPK6
Source: PLoS Pathog. 2015 Oct 20;11(10):e1005231. doi: 10.1371/journal.ppat.1005231 (PMC4617645; doi:10.1371/journal.ppat.1005231)
Supplement: S3 Fig — The same extracts as those in Fig 2E were separated on Phos-Tag SDS-PAGE. Proteins were detected by immunoblot assay with anti-myc antibody. (PPTX) [file ppat.1005231.s004.pptx]

## Slide 1
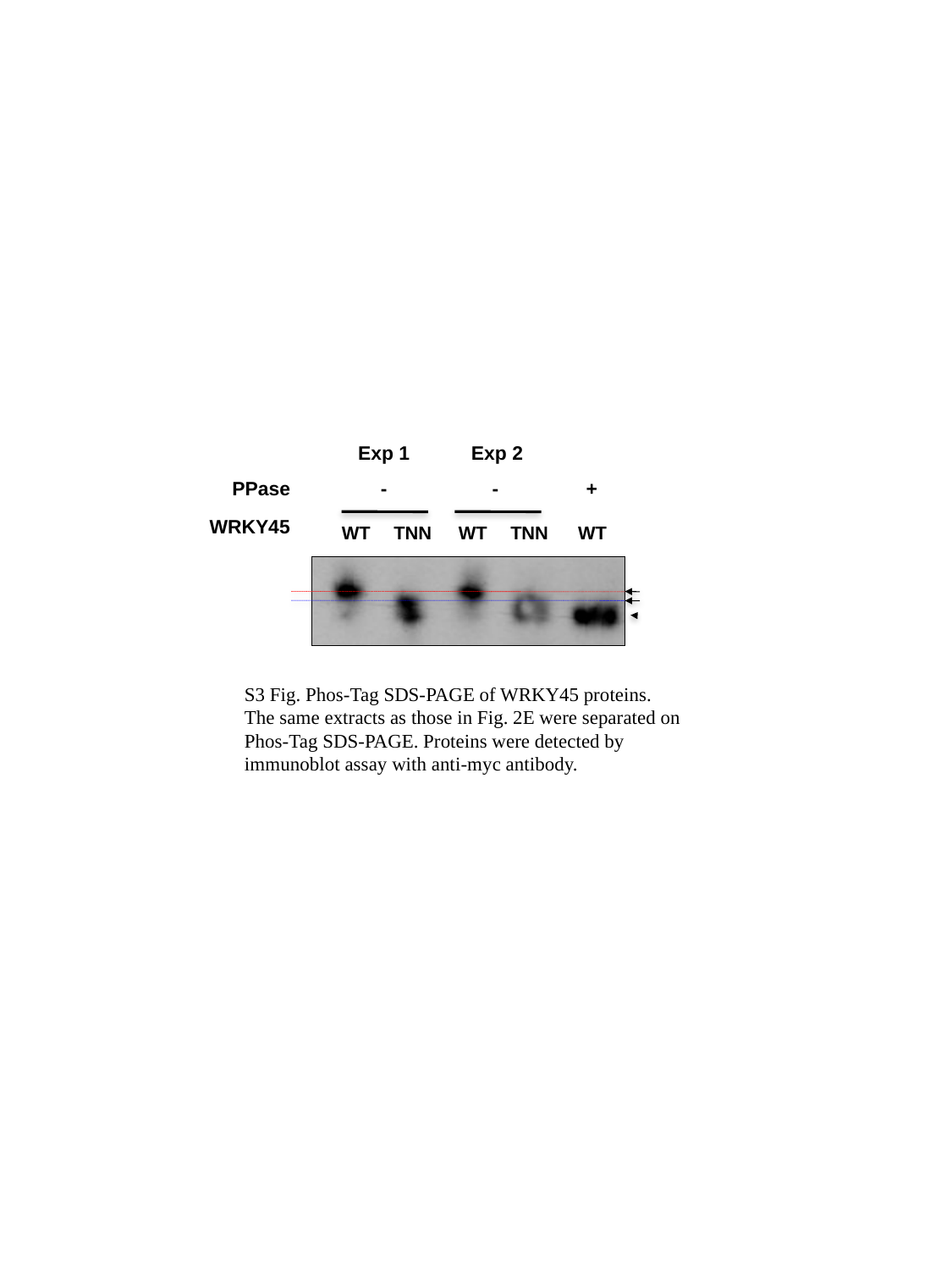

Exp 1
Exp 2
PPase
-
-
+
WRKY45
WT
TNN
WT
TNN
WT
S3 Fig. Phos-Tag SDS-PAGE of WRKY45 proteins.
The same extracts as those in Fig. 2E were separated on Phos-Tag SDS-PAGE. Proteins were detected by immunoblot assay with anti-myc antibody.
