# Supplementary material for: Abiotic Stresses Antagonize the Rice Defence Pathway through the Tyrosine-Dephosphorylation of OsMPK6
Source: PLoS Pathog. 2015 Oct 20;11(10):e1005231. doi: 10.1371/journal.ppat.1005231 (PMC4617645; doi:10.1371/journal.ppat.1005231)
Supplement: S4 Fig — Phosphorylation of WT and mutant forms (Y227D and T225A) of MBP-MPK6 proteins were assayed using [γ-32P]ATP as a substrate as described in Materials and Methods, CBB, loading control. (PPTX) [file ppat.1005231.s005.pptx]

## Slide 1
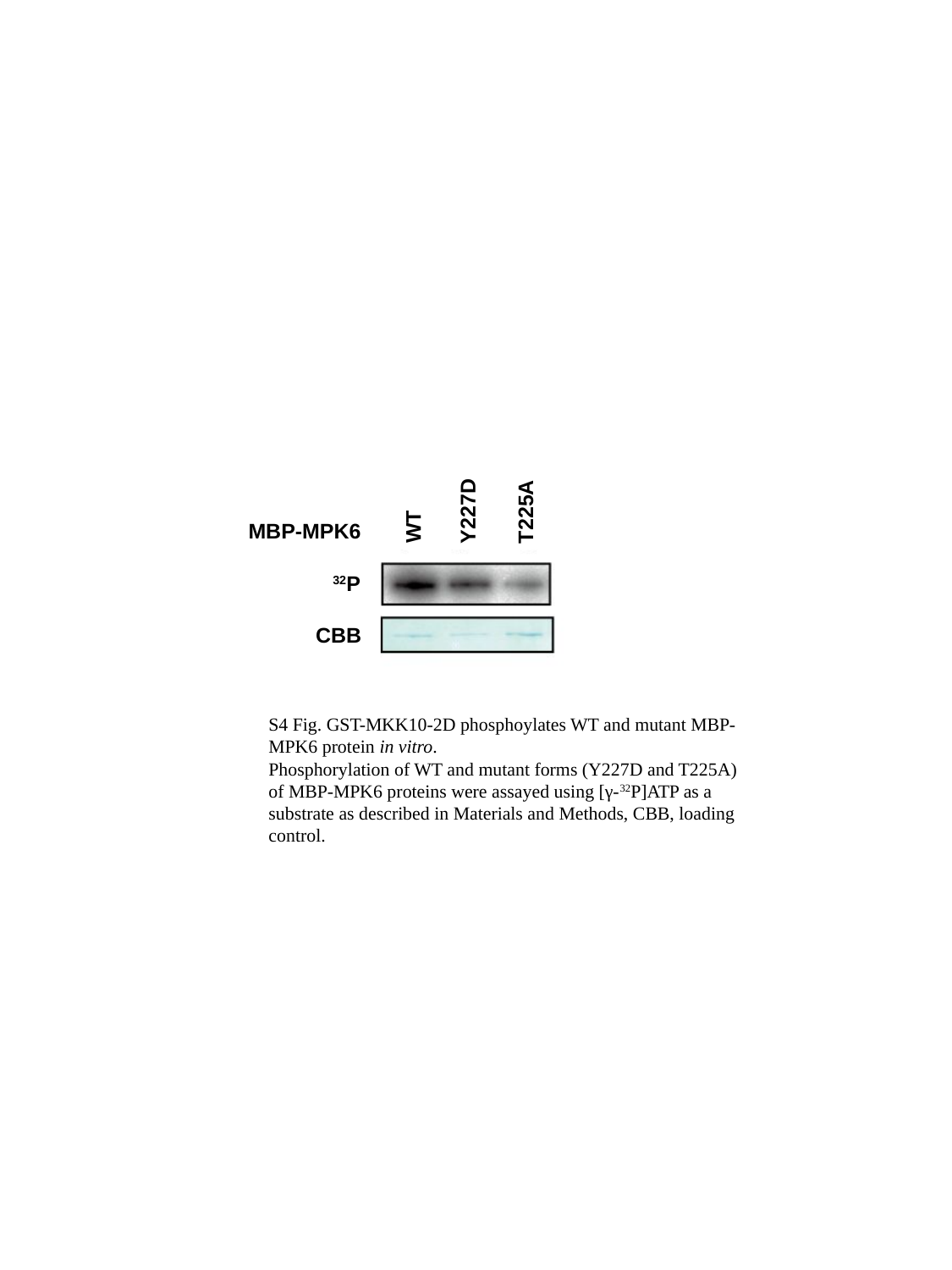

Y227D
T225A
WT
MBP-MPK6
32P
CBB
S4 Fig. GST-MKK10-2D phosphoylates WT and mutant MBP-MPK6 protein in vitro.
Phosphorylation of WT and mutant forms (Y227D and T225A) of MBP-MPK6 proteins were assayed using [γ-32P]ATP as a substrate as described in Materials and Methods, CBB, loading control.
