# Supplementary material for: Abiotic Stresses Antagonize the Rice Defence Pathway through the Tyrosine-Dephosphorylation of OsMPK6
Source: PLoS Pathog. 2015 Oct 20;11(10):e1005231. doi: 10.1371/journal.ppat.1005231 (PMC4617645; doi:10.1371/journal.ppat.1005231)
Supplement: S5 Fig — Transcript levels of WRKY45 and phenylalanine ammonia-lyase (PAL) genes (relative to that of ubiquitin 1) were determined by qRT-PCR. The same results with different scale are shown for WRKY45 in an inset. (PPTX) [file ppat.1005231.s006.pptx]

## Slide 1
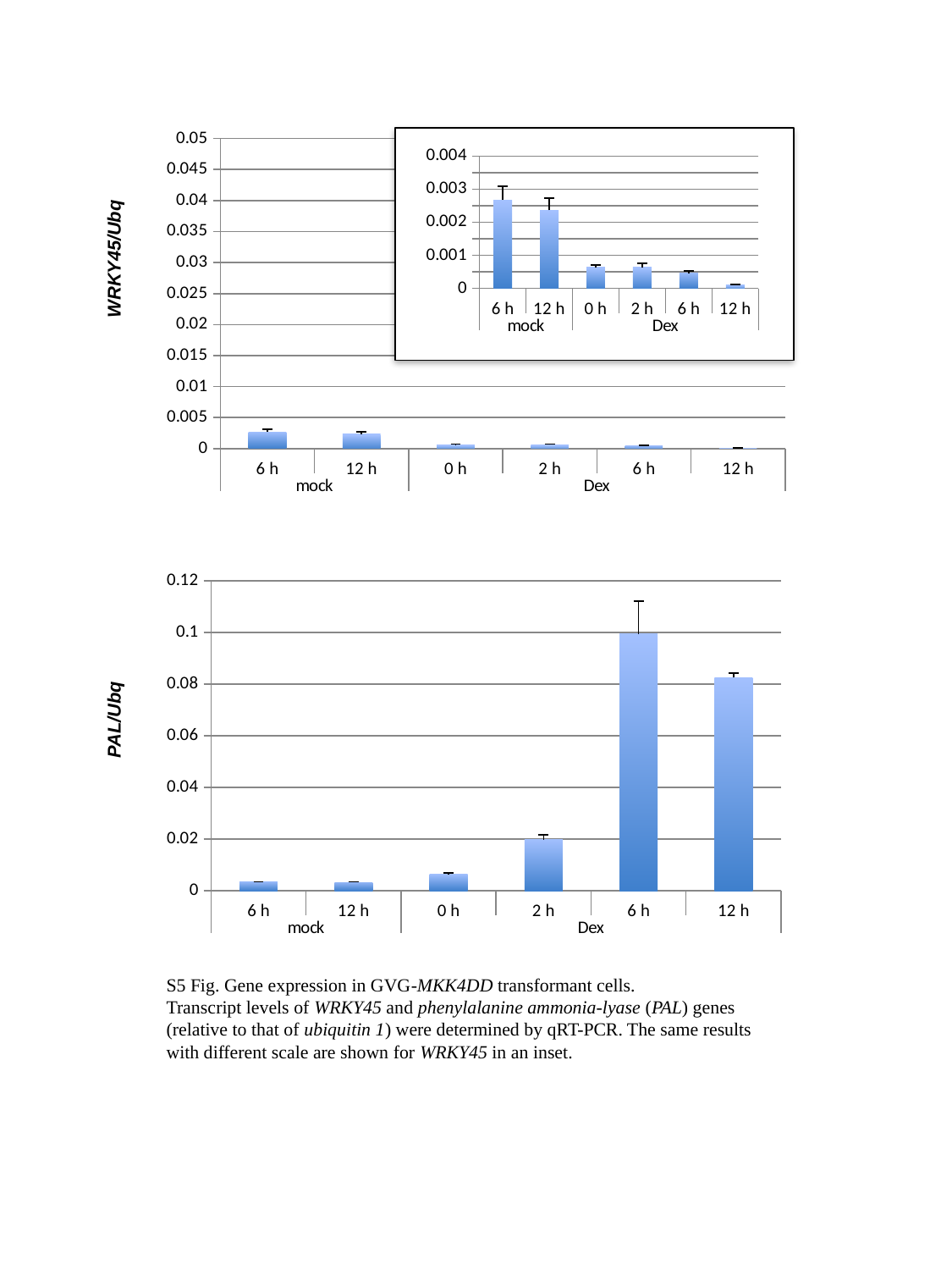

### Chart
| Category | W45/Ub |
|---|---|
| 6 h | 0.002677314 |
| 12 h | 0.002363272 |
| 0 h | 0.000637627 |
| 2 h | 0.0006398403 |
| 6 h | 0.0004571638 |
| 12 h | 0.0001107812 |
### Chart
| Category | W45/Ub |
|---|---|
| 6 h | 0.002677314 |
| 12 h | 0.002363272 |
| 0 h | 0.000637627 |
| 2 h | 0.0006398403 |
| 6 h | 0.0004571638 |
| 12 h | 0.0001107812 |WRKY45/Ubq
### Chart
| Category | PAL/Ub |
|---|---|
| 6 h | 0.003353777 |
| 12 h | 0.003239529 |
| 0 h | 0.00625836 |
| 2 h | 0.01977745 |
| 6 h | 0.09944193 |
| 12 h | 0.08246922 |PAL/Ubq
S5 Fig. Gene expression in GVG-MKK4DD transformant cells.
Transcript levels of WRKY45 and phenylalanine ammonia-lyase (PAL) genes (relative to that of ubiquitin 1) were determined by qRT-PCR. The same results with different scale are shown for WRKY45 in an inset.
