# Supplementary material for: Abiotic Stresses Antagonize the Rice Defence Pathway through the Tyrosine-Dephosphorylation of OsMPK6
Source: PLoS Pathog. 2015 Oct 20;11(10):e1005231. doi: 10.1371/journal.ppat.1005231 (PMC4617645; doi:10.1371/journal.ppat.1005231)
Supplement: S6 Fig — GVG-MKK10-2D line #14 was analyzed as in Fig 5A. (PPTX) [file ppat.1005231.s007.pptx]

## Slide 1
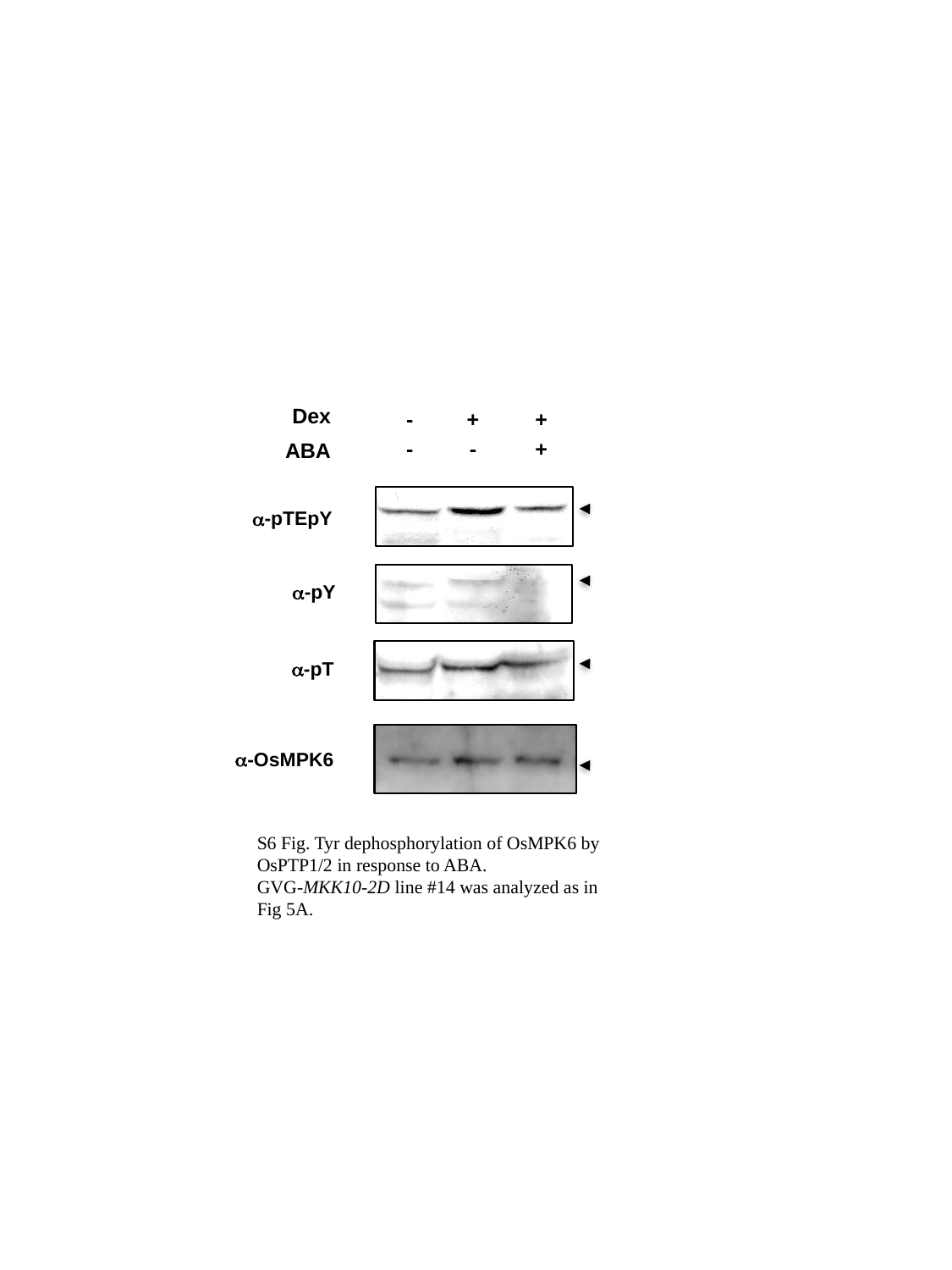

Dex
-
+
+
-
-
+
ABA
a-pTEpY
a-pY
a-pT
a-OsMPK6
S6 Fig. Tyr dephosphorylation of OsMPK6 by OsPTP1/2 in response to ABA.
GVG-MKK10-2D line #14 was analyzed as in Fig 5A.
