# Supplementary material for: Abiotic Stresses Antagonize the Rice Defence Pathway through the Tyrosine-Dephosphorylation of OsMPK6
Source: PLoS Pathog. 2015 Oct 20;11(10):e1005231. doi: 10.1371/journal.ppat.1005231 (PMC4617645; doi:10.1371/journal.ppat.1005231)
Supplement: S7 Fig — WT rice plants were treated with 1 mM SA and increasing concentrations of ABA, in the presence or absence of 2 mM vanadate or 50 μM Bay11-7082. *, P<0.05; **, P<0.001 (Student’s t-test). (PPTX) [file ppat.1005231.s008.pptx]

## Slide 1
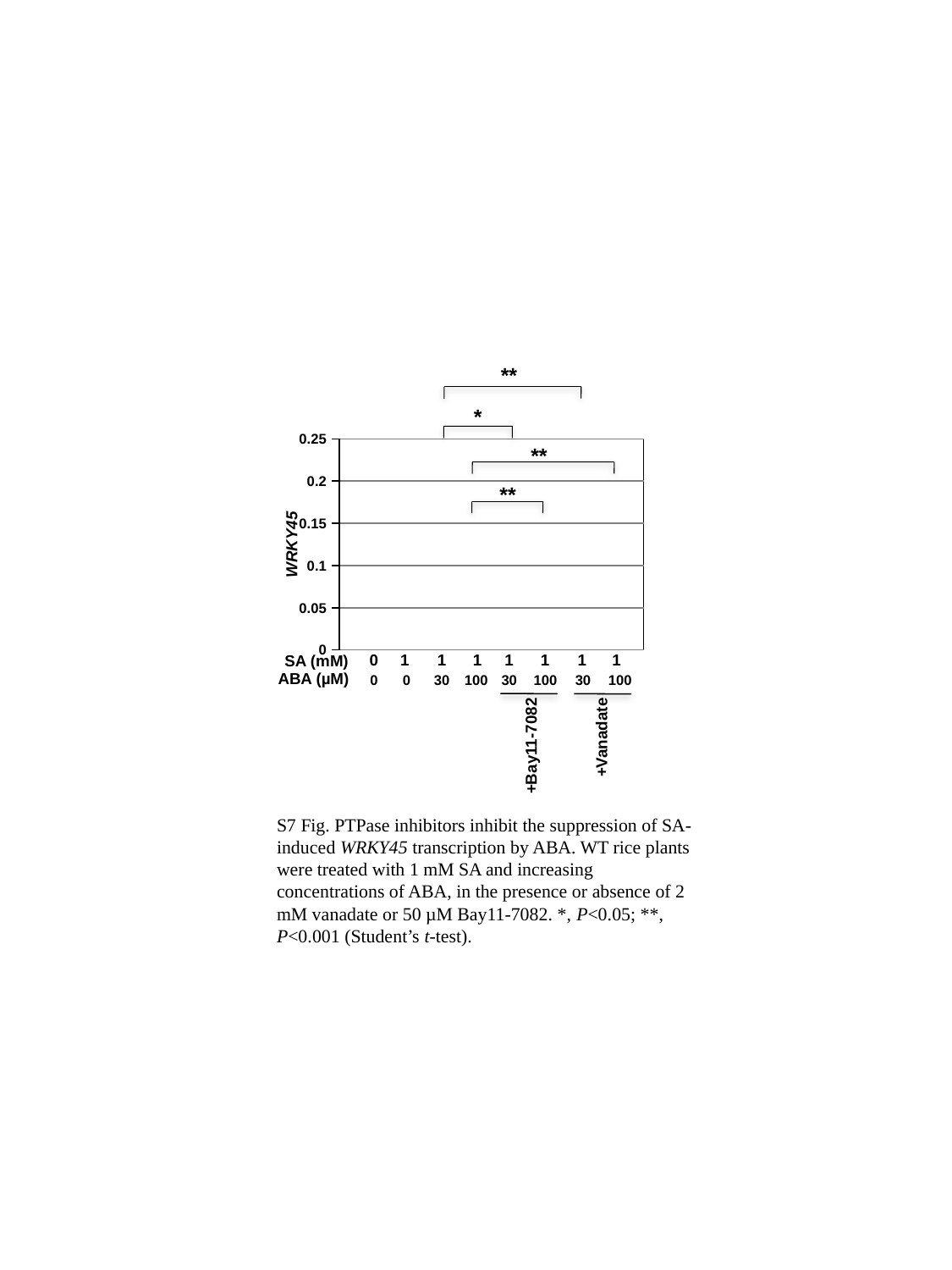

**
*
### Chart
| Category | WRKY45 |
|---|---|
| mock | 0.007416741 |
| SA | 0.1666624 |
| SA+30ABA (-1h) | 0.07721368 |
| SA+100ABA (-1h) | 0.06675406 |
| SA+30ABA (-1h)+50Bay | 0.1678215 |
| SA+100ABA (-1h)+50Bay | 0.1088188 |
| SA+30ABA (-1h)+2Vana | 0.1907825 |
| SA+100ABA (-1h)+2Vana | 0.1263067 |**
**
WRKY45
0
1
1
1
1
1
1
1
SA (mM)
ABA (µM)
0
0
30
100
30
100
30
100
+Vanadate
+Bay11-7082
S7 Fig. PTPase inhibitors inhibit the suppression of SA-induced WRKY45 transcription by ABA. WT rice plants were treated with 1 mM SA and increasing concentrations of ABA, in the presence or absence of 2 mM vanadate or 50 µM Bay11-7082. *, P<0.05; **, P<0.001 (Student’s t-test).
