# Supplementary material for: Abiotic Stresses Antagonize the Rice Defence Pathway through the Tyrosine-Dephosphorylation of OsMPK6
Source: PLoS Pathog. 2015 Oct 20;11(10):e1005231. doi: 10.1371/journal.ppat.1005231 (PMC4617645; doi:10.1371/journal.ppat.1005231)
Supplement: S8 Fig — Conserved residues are boxed. *, catalytically essential Cys residue. Active site signature [(I/V)HCXAGXXR(S/T)G] conserved in PTPs from all organisms is underlined. (PPTX) [file ppat.1005231.s009.pptx]

## Slide 1
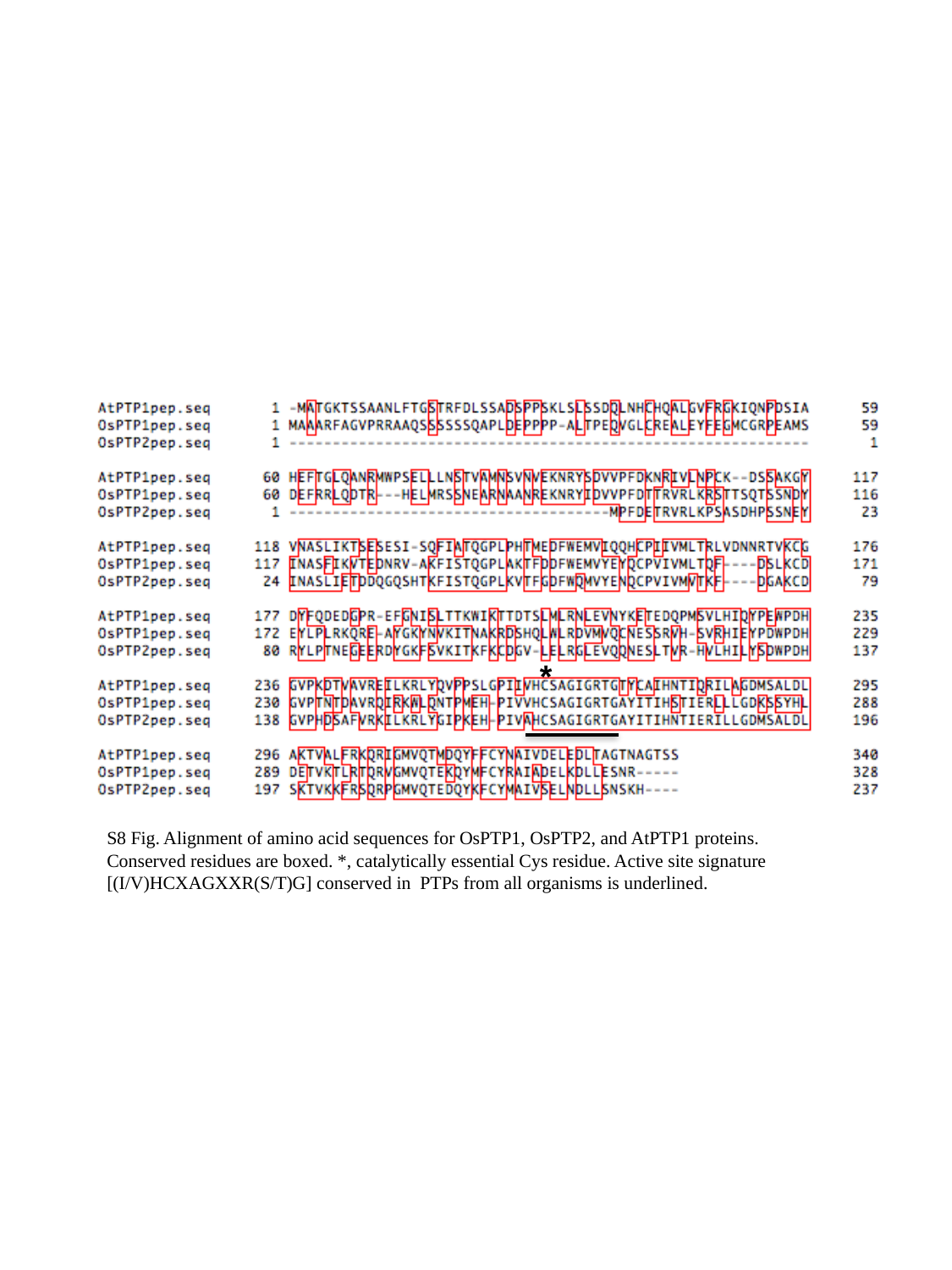

*
S8 Fig. Alignment of amino acid sequences for OsPTP1, OsPTP2, and AtPTP1 proteins.
Conserved residues are boxed. *, catalytically essential Cys residue. Active site signature [(I/V)HCXAGXXR(S/T)G] conserved in PTPs from all organisms is underlined.
