# Supplementary material for: Abiotic Stresses Antagonize the Rice Defence Pathway through the Tyrosine-Dephosphorylation of OsMPK6
Source: PLoS Pathog. 2015 Oct 20;11(10):e1005231. doi: 10.1371/journal.ppat.1005231 (PMC4617645; doi:10.1371/journal.ppat.1005231)
Supplement: S9 Fig — Transcript levels of OsPTP1 and -2 (relative to that of rice ubiquitin 1) in NB (WT) plants treated with 250 mM NaCl or 8°C for indicated day length were determined by RT-qPCR. (PPTX) [file ppat.1005231.s010.pptx]

## Slide 1
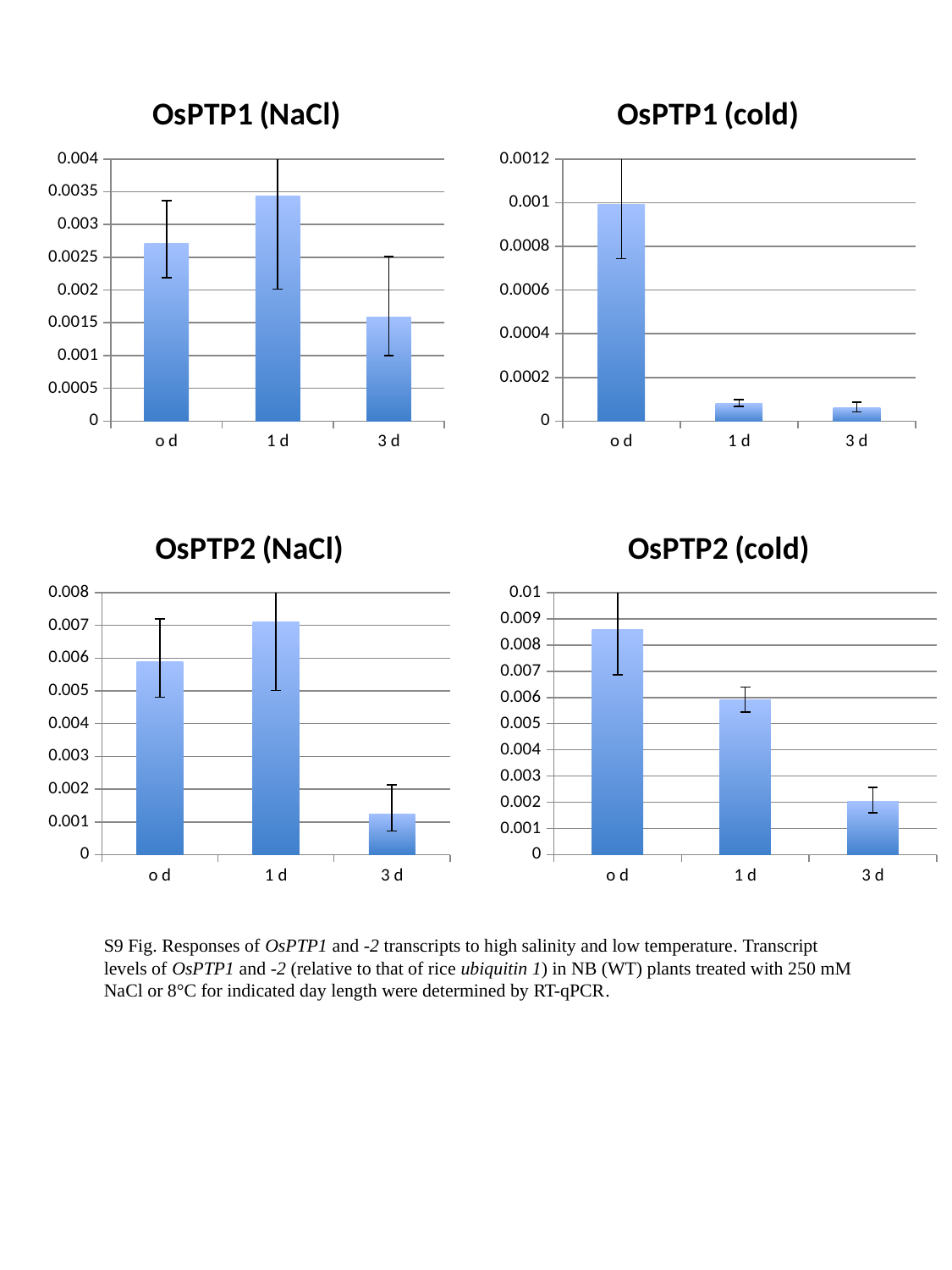

### Chart: OsPTP1 (NaCl)
| Category | PTP1 (NaCl) |
|---|---|
| o d | 0.002714685 |
| 1 d | 0.003436126 |
| 3 d | 0.001580941 |
### Chart: OsPTP1 (cold)
| Category | PTP1 (cold) |
|---|---|
| o d | 0.000990195 |
| 1 d | 8.0816e-05 |
| 3 d | 6.01949e-05 |
### Chart: OsPTP2 (NaCl)
| Category | PTP2 (NaCl) |
|---|---|
| o d | 0.005879871 |
| 1 d | 0.007114607 |
| 3 d | 0.001240383 |
### Chart: OsPTP2 (cold)
| Category | PTP2 (cold) |
|---|---|
| o d | 0.008578863 |
| 1 d | 0.005900288 |
| 3 d | 0.002029022 |S9 Fig. Responses of OsPTP1 and -2 transcripts to high salinity and low temperature. Transcript levels of OsPTP1 and -2 (relative to that of rice ubiquitin 1) in NB (WT) plants treated with 250 mM NaCl or 8°C for indicated day length were determined by RT-qPCR.
