# Supplementary material for: Abiotic Stresses Antagonize the Rice Defence Pathway through the Tyrosine-Dephosphorylation of OsMPK6
Source: PLoS Pathog. 2015 Oct 20;11(10):e1005231. doi: 10.1371/journal.ppat.1005231 (PMC4617645; doi:10.1371/journal.ppat.1005231)
Supplement: S10 Fig — Transcript levels of WRKY45 and OsNPR1 (relative to that of rice ubiquitin 1) in NB (WT) and PTP-wkd plants treated with 1 mM SA in the absence or presence of ABA (30 μM) were determined by RT-qPCR. Means of eight biological replicates are shown with SEM. ***, P<0.002 (Student’s t-test). (PPTX) [file ppat.1005231.s011.pptx]

## Slide 1
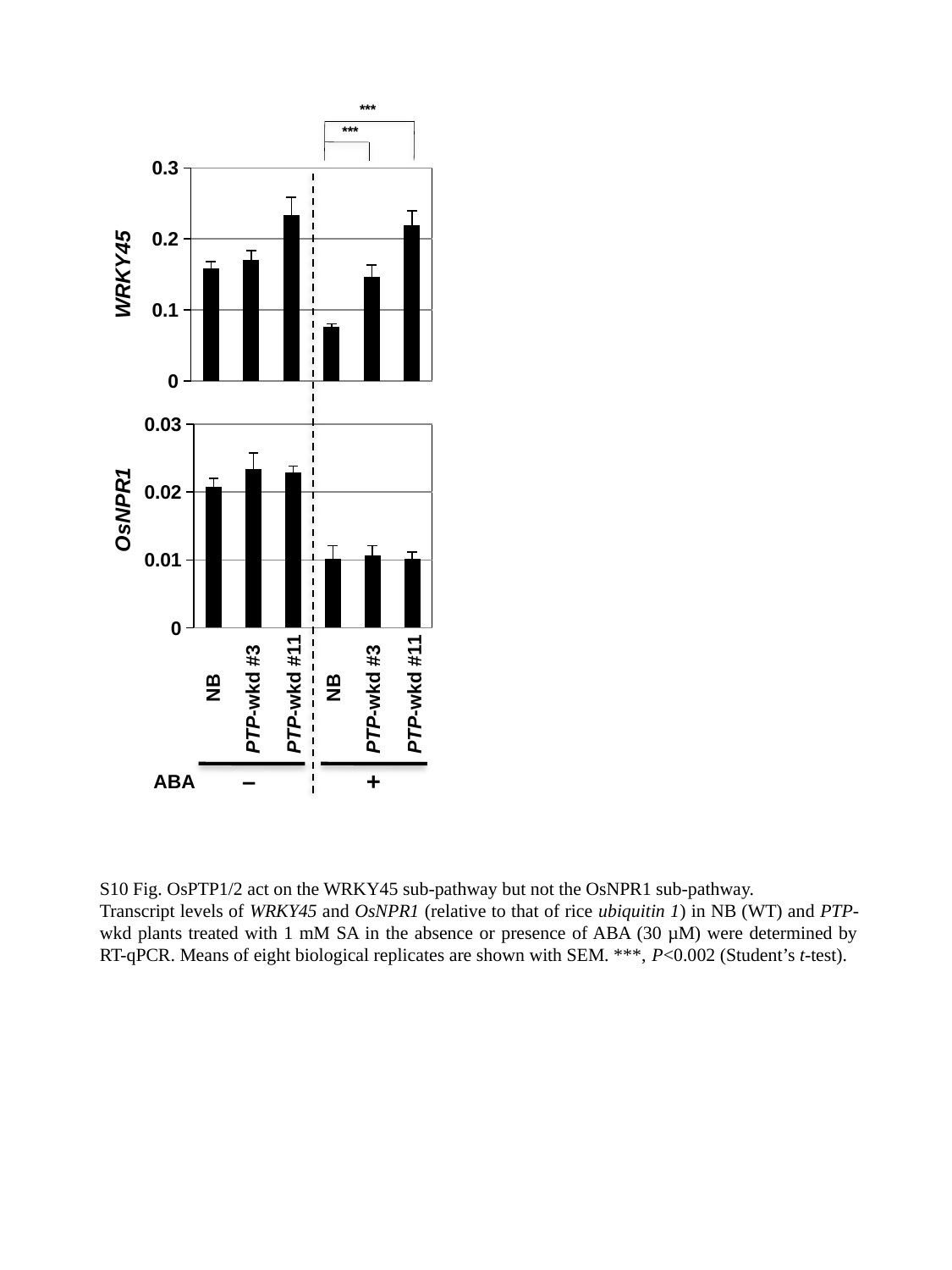

***
***
### Chart
| Category | WRKY45 |
|---|---|
| NB | 0.158622475 |
| 3A34 | 0.1701120375 |
| 11C18 | 0.23393885 |
| NB | 0.075788495 |
| 3A34 | 0.146714855 |
| 11C18 | 0.21966605 |WRKY45
### Chart
| Category | NPR1 |
|---|---|
| NB | 0.020780855 |
| #3 | 0.02336773 |
| #11 | 0.02283366875 |
| NB | 0.010159528 |
| #3 | 0.0106566845 |
| #11 | 0.010133197375 |OsNPR1
NB
PTP-wkd #11
PTP-wkd #3
NB
PTP-wkd #11
PTP-wkd #3
–
+
ABA
S10 Fig. OsPTP1/2 act on the WRKY45 sub-pathway but not the OsNPR1 sub-pathway.
Transcript levels of WRKY45 and OsNPR1 (relative to that of rice ubiquitin 1) in NB (WT) and PTP-wkd plants treated with 1 mM SA in the absence or presence of ABA (30 µM) were determined by RT-qPCR. Means of eight biological replicates are shown with SEM. ***, P<0.002 (Student’s t-test).
